# Coordinating brain-distributed network activities in memory resistant to extinction

**DOI:** 10.1016/j.cell.2023.12.018

**Published:** 2024-01-18

**Authors:** Charlie J. Clarke-Williams, Vítor Lopes-dos-Santos, Laura Lefèvre, Demi Brizee, Adrien A. Causse, Roman Rothaermel, Katja Hartwich, Pavel V. Perestenko, Robert Toth, Colin G. McNamara, Andrew Sharott, David Dupret

**Affiliations:** Medical Research Council Brain Network Dynamics Unit, Nuffield Department of Clinical Neurosciences, University of Oxford, Oxford, OX1 3TH, UK

## Abstract

Certain memories resist extinction to continue invigorating maladaptive actions. The robustness of these memories could depend on their widely distributed implementation across populations of neurons in multiple brain regions. However, how dispersed neuronal activities are collectively organized to underpin a persistent memory-guided behavior remains unknown. To investigate this, we simultaneously monitored the prefrontal cortex, nucleus accumbens, amygdala, hippocampus, and ventral tegmental area (VTA) of the mouse brain from initial recall to post-extinction renewal of a memory involving cocaine experience. We uncover a higher-order pattern of short-lived beta-frequency (15–25 Hz) activities that are transiently coordinated across these networks during memory retrieval. The output of a divergent pathway from upstream VTA glutamatergic neurons, paced by a slower (4-Hz) oscillation, actuates this multi-network beta-band coactivation, which closed-loop phase-informed suppression prevented renewal of cocaine-biased behavior. Binding brain-distributed neural activities in this temporally structured manner may constitute an organizational principle of robust memory expression.

## Introduction

Memories typically support adaptation to the world we live in, allowing past experience to inform choices that are most appropriate to ongoing circumstances ^1^. However, not all memories adequately serve behavior this way. Notably, drugs of abuse such as cocaine produce powerful memories that associate drug experience with surrounding information, linking spatial context, discrete stimuli and behavioral actions ^2^. Re-exposure to drug-associated stimuli evokes the memory recall of experiences involving cocaine, either directly or through indirectly related events. Thus, retrieval of cocaine-paired memories can invigorate drug-biased behavior even in contexts where the drug was never encountered. In such contexts, behavioral patterns of drug-biased actions eventually extinguish. But despite extinction, the chain of events linked to cocaine is not forgotten and drug-paired signals can elicit the recovery of cocaine-biased behavior; a process known as renewal ^3–8^. This cocaine-paired memory retrieval, from initial recall to post-extinction renewal, increases the likelihood of further maladaptation. Previous studies have identified how cocaine alters molecular signaling, synaptic plasticity and cellular activity (e.g., ^9–15^). However, the brain-network-level mechanisms of cocaine-paired memory retrieval remain uncertain.

A central difficulty in identifying the network mechanisms of cocaine-paired memory retrieval could pertain to the distributed nature of this process, recruiting neurons from not one but multiple regions. Previous studies focusing on one brain region, or pairs of at most, have reported that important nodes in this distributed organization include the medial prefrontal cortex (PFC), the nucleus accumbens (NAc), the amygdala (Amy) and the hippocampus (Hpc) ^16–27^. Neural dynamics in all these regions support memory-guided behaviors ^28–35^. In addition, the ventral tegmental area (VTA) has consistently been described as a central region for internal processing of reward/drug experience and memory ^36–47^. The VTA circuitry involves diverse cell types genetically defined to express dopamine, glutamate, GABA, or a combination of these neurotransmitters ^48,49^. Whether (and if so how) the brain ultimately coordinates the neural activities distributed across these regions into a coherent, larger-scale (meta-) ensemble of neurons that collectively influence behavior remains unknown. *In vivo* electrophysiological recordings and theoretical work have highlighted that network oscillations report effective communication channels between distributed populations of neurons ^50–57^. Accordingly, here we investigate whether the dynamic retrieval of cocaine-paired memory involves selective patterns of network oscillatory activities that functionally couple neurons dispersed across multiple regions, from initial recall to post-extinction renewal. Under this scenario, our overarching objective is to identify the cellular implementation of such cross-network coordination, leveraging this knowledge to deploy fine-grained feedback interventions for preventing re-emergence of cocaine-biased behavior.

To proceed, we developed a paradigm that models stages of cocaine-paired memory in mice, while performing quintuple-brain-region recordings of network oscillations and neuronal ensembles from PFC, NAc, Amy, CA1 Hpc and VTA. We designed an unsupervised analysis framework to detect and track activity patterns of multiregional coordination (“barcodes”). This unveiled a higher-order pattern of short-lived beta-band (15–25 Hz) neural co-activities during memory recall and renewal. This cross-network coordination is paced by the phase of a slower (4-Hz) oscillation in VTA, involving VTA glutamatergic neurons that project to the other regions. Activating VTA glutamatergic cells entrained neuronal spiking and induced cross-regional beta-band responses; orientating behavior towards place-cue stimuli paired with animal position-controlled 4Hz-self-stimulation. Real-time closed-loop silencing of VTA glutamatergic neurons at their 4-Hz preferred phase disrupted cross-network beta-band coordination and prevented renewal of cocaine-biased behavior. Applying 4-Hz phase-informed silencing to the subset of VTA glutamatergic/dopaminergic neurons was sufficient to disrupt beta-band coordination and block memory renewal. These findings identify the oscillatory structure and anatomical pathway of a neural pattern that transiently binds neuronal populations distributed across brain regions in robust memory expression.

## Results

### Modelling behavioral dynamics of cocaine-paired memory retrieval

**Figure 1:**
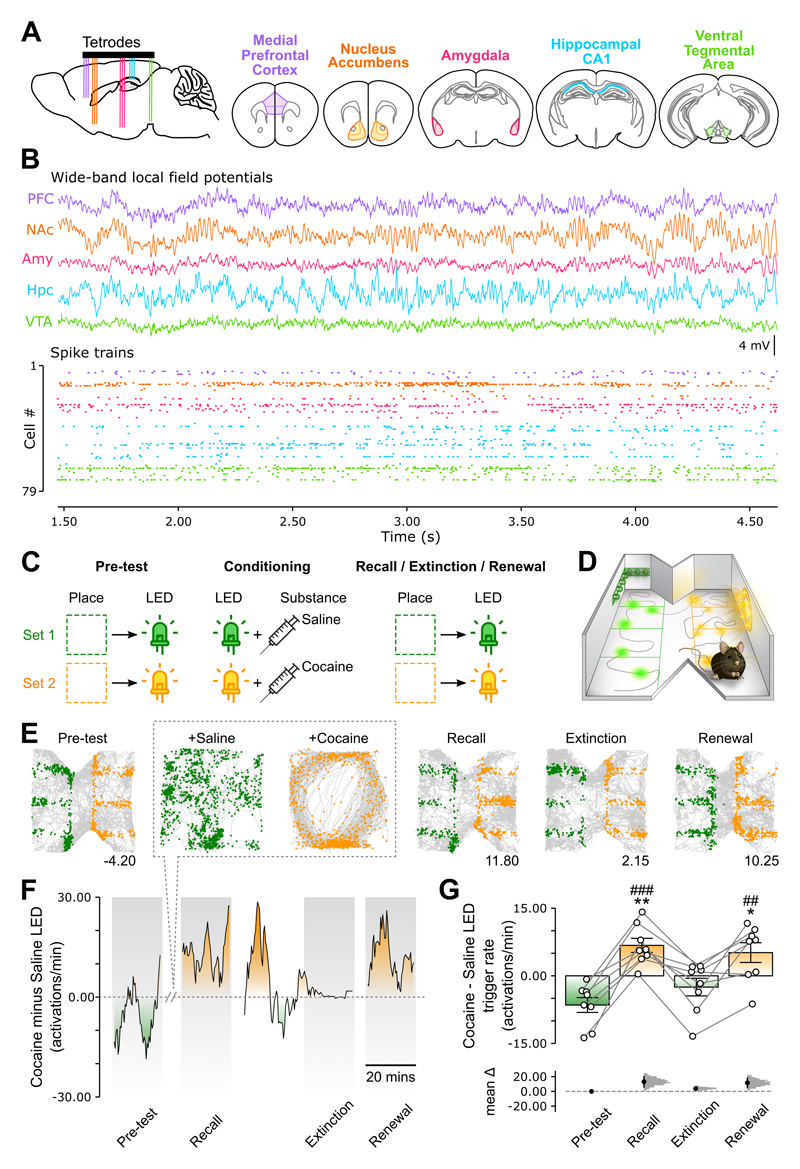
Recording distributed brain networks in a cocaine conditioned cue-place preference task. **(A)** Quintuple-brain-region multichannel recording layout. PFC, NAc, Amy, dorsal CA1 Hpc and VTA (*left:* schematic sagittal section; *right:* corresponding coronal sections) targeted with independently moveable tetrodes. **(B)** Example wide-band signals (top: raw LFP traces) simultaneously recorded with individual neuron spikes (bottom: raster plot of color-coded spike trains; one cell per row). **(C,D)** Conditioned cue-place preference task layout (**C**) and its test enclosure (**D**) with a place-controlled LED activation system for mice to acquire two sets of place-LED-substance associations (set 1 saline versus set 2 cocaine). Mouse preference for activating cocaine- versus saline-paired LEDs quantified as a measure of cocaine-biased behavior. (**E)** Example animal paths (gray). Green and orange dots: animal’s position during saline- and cocaine-paired LED activations, respectively. Numbers indicate difference in cocaine- minus saline-paired LED activations per minute (negative: preferred saline-paired LED activation; positive: preferred cocaine-paired LED activation). Note the mouse expressed cocaine-paired LED biases during recall and renewal. (**F)** Example time course of cocaine-paired LED activation bias across tests. During recall, the mouse reverted its initial (pre-test) preference for the cocaine-paired LED; this diminished during extinction but re-occurred during renewal. (**G)** The dataset is represented using a difference estimation plot (see STAR Methods) to visualize the effect size for changes in cocaine-biased behavior across tests. *Top*: raw data points, with each set of four connected points reporting the difference between cocaine-minus saline-paired LED activation for one mouse in each stage; bar charts: average (mean±SEM) LED activation preference over animals. *Bottom:* corresponding effect size, using the mean difference estimation for each stage compared to Pre-test. *Black-dot*, mean difference; *filled-curve:* distribution of mean differences; *black-lines*, 95% confidence interval; ### p<0.001; ## and ** p<0.01; # and * p<0.05; repeated ANOVA for pairwise stage-stage (#: vs pre-test; *: vs extinction) interactions, with Bonferroni correction for multiple comparisons.

To explore the network-level signature of cocaine-related memory, we developed a paradigm that models the dynamic expression of drug-biased behavior while permitting quintuple-brain-region recordings of both local field potentials (LFPs) and ensemble spike trains, simultaneously from PFC, NAc, Amy, CA1 Hpc and VTA (Figures 1A,B and S1A,B). Our task leverages the conditioned place preference paradigm, ^27,58–60^ measuring the propensity of the animal to self-activate a short-lived sensory cue (conditioned stimulus) by visiting a discrete location not itself directly linked to drug experience (Figure 1C-G). This conditioned cue-place preference task uses a five-stage layout spanning multiple days, through which mice acquire an indirect association between their ability to trigger an LED in one environment, and an LED-paired cocaine experience in another environment.

In the first stage (Figure 1C; “pre-test”; 2 days), mice explored a bow-tie-shaped arena (“test enclosure”; Figure 1D) that contained two opposite zones of four unmarked quadrants. Using a place-controlled LED activation system, this stage allowed mice to learn two sets of place-LED associations where they could momentarily trigger one of two distinct LED wall-displays (green L-shape versus orange circle^61,62^; mean LED activity duration = 392 ms; interquartile range = 42–634 ms) by moving within their corresponding zone (Figure 1C,D). We defined the last twenty minutes of this stage as the pre-test session, where we identified the initial (pre-conditioning) preference that each animal had for one LED while in the test enclosure, and to log the corresponding behavioral pattern of place-controlled LED activations.

In the second stage (Figure 1C; “conditioning”; 3 days), mice associated each LED with either saline or cocaine in another, square-shaped arena (“conditioning enclosure”). Despite being spatially distinct and not equipped with the place-LED controller, this enclosure shared some contextual features with the test enclosure. On each conditioning day, having received a saline injection, the animal explored this enclosure where the preferred LED now automatically flickered, emulating the behavioral pre-test LED activation pattern. The animal then received cocaine and re-explored the conditioning enclosure where the non-preferred LED would automatically emulate the behavioral pre-test activation pattern (Figures 1E and S1C).

On the day following the last conditioning session, mice returned to the test enclosure (containing the place-LED controller) for the remaining three stages: “recall,” “extinction,” and “renewal” (Figure 1C; 1 day). Throughout these tests, we measured the animal’s propensity to activate each LED. During recall, mice inverted their pre-test preference for the cocaine-paired LED (Figure 1E-G). This post-conditioning inversion was unlikely explained by a LED novelty bias, since the place-LED preference initially expressed by mice remained similar across the 2-day pre-test stage (Figure S1D). This cocaine-conditioned LED preference evidenced that mice inferred across enclosures a second-order relationship between their ability to directly trigger each LED and their separate pairing with a substance.

Contextual alterations during transitions from stimulus-cued drug availability to unavailability decrease drug-motivated behavior: a phenomenon known as extinction. However, following extinction, re-exposure to discrete stimuli present within the drug-associated context revives drug-oriented responses: a phenomenon known as renewal ^3,8,63^. We modelled these phenomena in the final two tests. We altered the test enclosure to un-match the environmental features of the extinction stage to those experienced in pre-test and recall (i.e., different arena floor and no background white noise). Over time, the cocaine-paired LED preference decayed (Figure 1F). We defined the last twenty minutes of this session as extinction to permit comparable analyses with the other tests. For the renewal stage, the test enclosure was re-installed with all its original contextual features and cocaine-biased behavior immediately recovered (Figure 1E-G). Thus, mice renewed an extinguished association between their ability to activate an LED and a separate cocaine experience. Applying Factor Analysis to assess covariance between measures of zone-biased exploration further suggested that a common factor underlaid recall and renewal (Figure S1E,F). Combining this conditioned cue-place preference task with electrophysiological recordings allowed determining whether specific patterns of brain network activity report cocaine-paired memory retrieval.

### Multiregional beta-band coordination during memory retrieval

**Figure 2:**
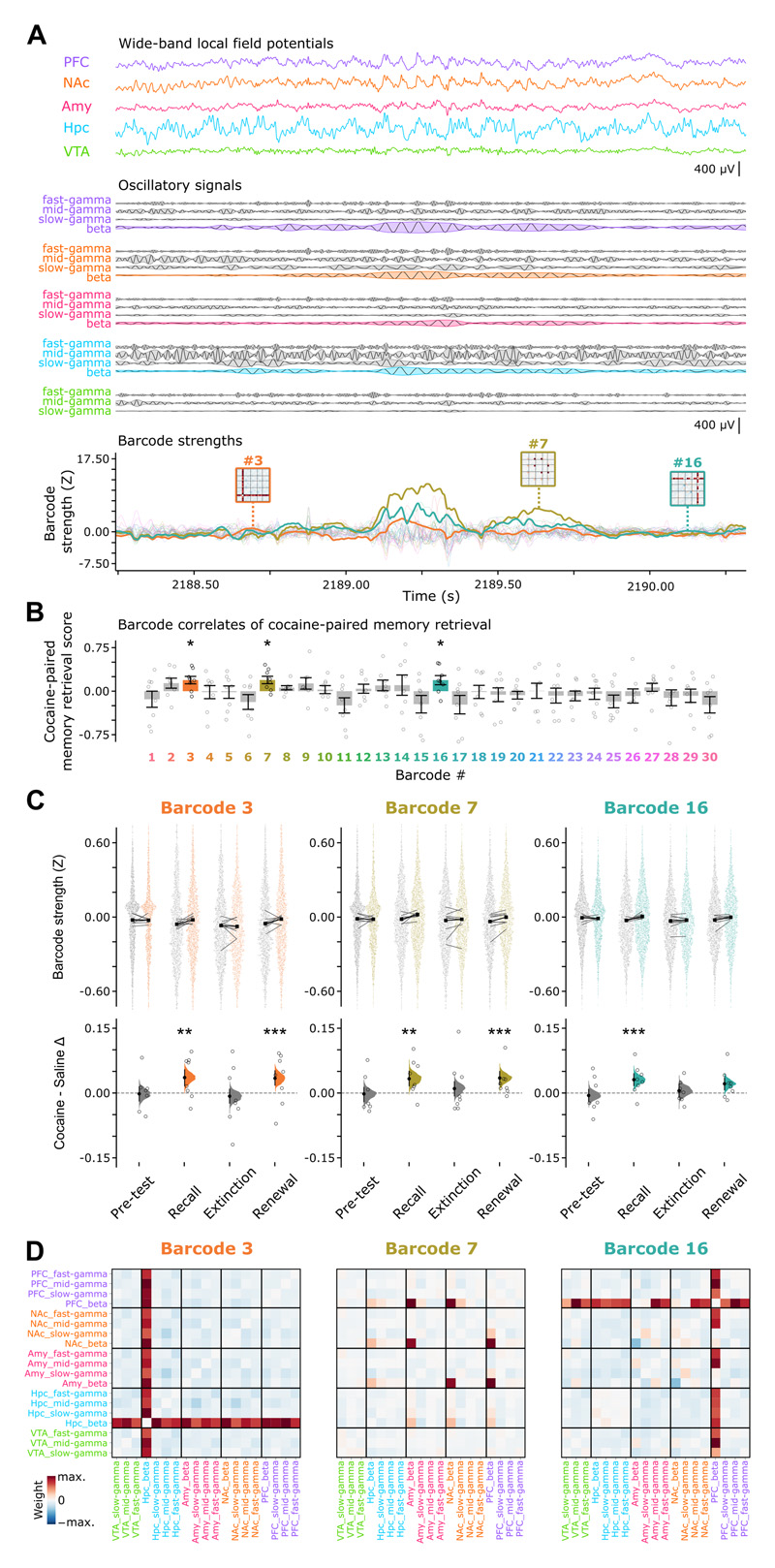
Cross-network beta-band activity barcodes report memory retrieval. (**A**) Example LFP traces (*top*) and their high-frequency (12–125 Hz) signals (*middle;* with instantaneous amplitudes as filled envelopes; color-coded, beta-band; gray, slow, mid, and fast gamma-bands). Corresponding cross-regional barcode strength time courses (*bottom; color-coded* and *bold*, barcodes # 3, 7 and 16; *fainter lines*, all other barcodes). See also Figure S2. (**B)** Barcode cocaine-paired memory retrieval scores. A higher score indicates that the barcode strength reflects memory recall and renewal (see Figure S2K). *Data points:* individual mice; *bar charts:* mean±SEM over mice. * p < 0.05: 1-sample t-test. (**C**) Cross-test changes in cocaine minus saline zone modulation strength for the three barcodes with the highest memory retrieval scores (**B**). Top: beeswarm plots of barcode strength during exploration (speed > 2 cm s^-1^; 1-s windows) of saline- (gray dots) versus cocaine- (colored dots) paired LED activation zones across tests; *gray lines:* mean zone strengths for each animal; *black squares/lines:* distribution average. Bottom: corresponding mean difference. *black-dot:* mean difference estimate; *filled-curve:* distribution of mean difference estimates; *black-lines:* 95% confidence interval; *open circles:* animal mean differences. *** p < 0.001; ** p < 0.01: permutation test, with Bonferroni correction for multiple comparisons (n = 4 stages). (**D)** Barcode visualization. Note that beta-band signal interactions dominate these barcodes.

We developed an unsupervised analysis framework to identify neural patterns of higher-order coordination, which we refer to as cross-network activity “barcodes.” We extracted the region-tailored series of oscillatory signals composing each brain network LFPs (Figures 2A and S2A,B). To focus on transient activities ^50^, we used signals spanning the 12–125 Hz frequency range and computed the time course of their instantaneous amplitudes (Figure 2A). These amplitude signals allowed detecting moment-by-moment co-occurrence of short-lived activities, which were sampled over all animals to detect the barcodes (Figure S2C-I). Having validated the consistency of barcode detection across animals (Figure S2I,J), we computed each barcode strength over the time-course of the multiregional LFPs throughout task stages (Figures 2A and S2G). With this, we computed for each barcode a cocaine-paired memory retrieval score to report the effect size for the difference in cocaine-versus-saline LED-zone strength with respect to changes in LED preference from pre-test to recall, extinction, and renewal (Figures 2B and S2K).

Remarkably, the barcodes significantly stronger in the cocaine-paired LED zone during recall and renewal (i.e., barcodes #3, #7 and #16; Figure 2C) all featured beta-band (15–25 Hz) signals (Figures 2A,D and S3A). We confirmed this enhanced beta-band barcode strength with a linear mixed model that regressed out the effects of individual animals (barcode #3: p < 0.05; barcodes #7, #16: p < 0.01; mixed-effects ANOVA model for zone + animal ~ strength, with Bonferroni correction for multiple comparisons (30 barcodes x 4 stages = 120)). A mixed-effects ANCOVA model further regressing out speed showed similar results. This speed- and animal-variation-accounted analysis revealed a significant cocaine-LED zone-selective enhancement of beta barcodes during recall and their significant reduction in the post-recall session that progressed towards extinction when the learnt drug-LED association was finally extinguished (recall: barcodes #3, #7 and #16: p < 0.05; extinction: barcodes #3, #7: p < 0.001; barcode #16: p < 0.05; mixed-effects ANCOVA models for zone + animal + speed ~ strength, with Bonferroni correction for multiple comparisons; and Figure S3B-D). When including theta-band signals in the barcode analysis, beta-band signals continued to feature most of the barcodes with significantly stronger expression in the cocaine-paired LED zone during recall and renewal (Figure S3E,F). Moreover, the amplitude of beta-band signals consistently increased in all regions during recall and renewal (i.e., stronger beta amplitudes in cocaine- than in saline-paired LED zones), a cross-regional change neither observed for the lower (theta) nor higher (gamma) frequency bands (Figure S3G). These results showed that a cross-network activity pattern in the beta-frequency range reports cocaine-paired memory expression.

### Cross-network beta-band modulation of neuronal spiking

**Figure 3:**
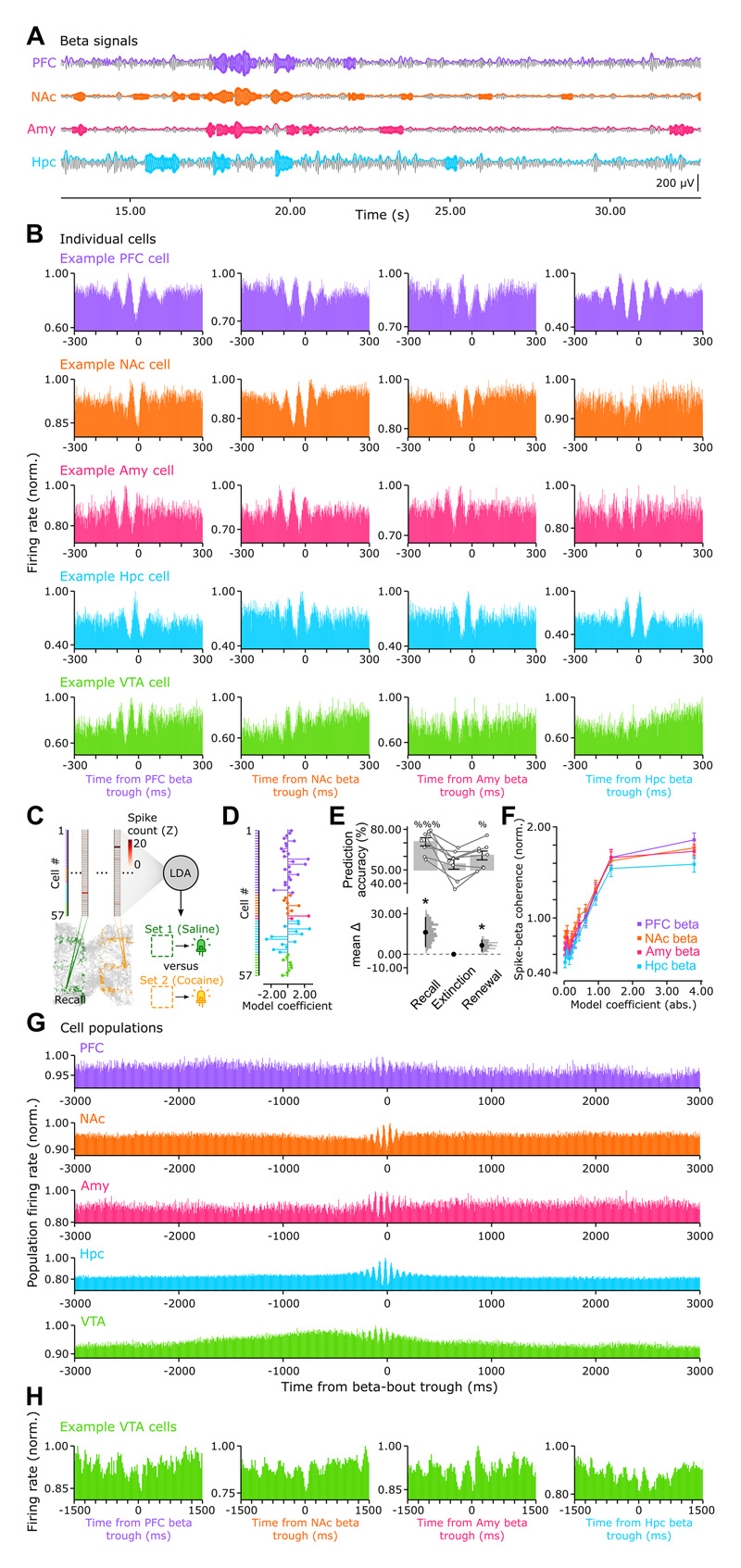
Local neuronal spiking is coupled to cross-regional beta oscillations. (**A)** Example beta-band signals from PFC, NAc, Amy and Hpc (gray lines) with instantaneous amplitude envelopes (colored lines) and high-amplitude bouts (colored filled parts). (**B**) Example beta-modulated cells for each region (one cell per row), with firing relative to beta-bout troughs (one trough per bout) in either PFC, NAc, Amy or Hpc CA1 (one region beta-trough per column; 1.5-ms bins). Note that triggering neuronal spiking by beta troughs (separated by at least 250 ms) shows at least three peaks of rhythmic firing modulation matching beta frequency. (**C-F**) Relationship between neuronal instantaneous spiking, beta-band coupling, and place-LED-substance association assessed using linear discriminant analysis models (see also Figure S4B-D). Each model was fitted to identify the active place-LED (set 1 versus set 2) from the ongoing population vectors of spike counts (100-ms windows) during recall (**C**), yielding a weight vector (example shown in **D**) with individual cell contribution to population decoding. Each model was applied in recall, extinction and renewal (**E**; * p < 0.05, paired mean difference distribution estimation versus extinction, with Bonferroni correction for multiple comparisons; n = 2 stages). Cells with stronger contribution to population decoding exhibited higher spike-beta coherence (**F**). **(G)** Time course of PFC, NAc, Amy, Hpc CA1 and VTA population firing relative to beta-bout troughs (NAc reference; 1.5-ms time bins). **(H)** Example VTA cells with firing relative to troughs of PFC, NAc, Amy or Hpc CA1 beta signals (25-ms bins). Note the rhythmic fluctuation of VTA spiking with a ~250-ms timescale.

We next investigated whether the identified beta-band network activity related to neuronal firing modulation. We detected bouts of high-amplitude beta in each region (Figure 3A; mean and standard deviation of beta-bout durations: 420.61±21.50; 491.80±14.03; 438.68±7.44; 481.10±16.67 ms for PFC, NAc, Amy, and Hpc, respectively). By using in turn each regional beta signal as a reference, we observed temporal modulation of individual neuron spiking with respect to beta-bout troughs (Figure 3B; using a single trough per beta-bout, ensuring trough separation by at least 250 ms; see also Figure S4A). Task-relevant information was tuned to beta-paced activity. We trained linear models (Linear Discriminant Analysis) on population vectors of spike discharge during recall to predict the active place-LED set (Figure 3C; mean number of neurons per model, 64.13; interquartile range, 51.00–83.50). Each model consisted of a cell-wise vector of coefficients whose magnitudes reflected the decoding contribution of each neuron (Figure 3D). Population decoding was significantly above chance during recall (Figure 3E; p = 2.393x10^-4^, 1-sample t-test), identifying with up to 79.31% accuracy the ongoing place-LED set (interquartile range 64.91–76.96%; median 74.06%; chance level of 50%). Decoding accuracy remained above chance in the high behavioral cocaine-LED preference windows during the post-recall session progressing towards extinction, when decoding then fell to chance level as the learnt place-LED association was extinguished (Figures S3B and S4B,C). Recall-trained models performed again above chance during renewal with cocaine-LED preference reexpression (Figure 3E; p = 1.364x10^-2^, 1-sample t-test; see also Figure S4D). This suggested that the preservation of spiking activity structure from recall to renewal related to the mnemonic features of cocaine experience, and not to a contextual (sensory) distinction. We further found that the decoding contribution of individual cells positively correlated with their spiking coherence to cross-regional beta signals (Figure 3F; PFC: r = 0.405, p = 4.19x10^-61^; NAc: r = 0.335, p = 2.75x10^-41^; Amy: r = 0.360, p = 9.26x10^-48^; Hpc: r = 0.360, p = 5.87x10^-48^). Beta-paced neural activities thus appeared relevant to the goal-directed behavior in this task, and we sought to identify the biological implementation of this cross-network coordination.

By examining the population firing time course of each region, we observed that VTA cells exhibited a marked increase in firing before beta bouts (Figure 3G). When triggering individual VTA cell spiking with respect to the trough of the beta signals of the other regions, VTA firing further showed modulation over a slower (~250-ms window, i.e., ~ 4-Hz) timescale (Figure 3H). The VTA has consistently been reported as central to reward/drug-oriented behavior and memory ^36–46^. This temporally-structured firing relationship suggested that the VTA can organize the transient beta-paced activities distributed across the other brain regions.

### VTA 4-Hz oscillations pace distributed beta-band network activities

**Figure 4:**
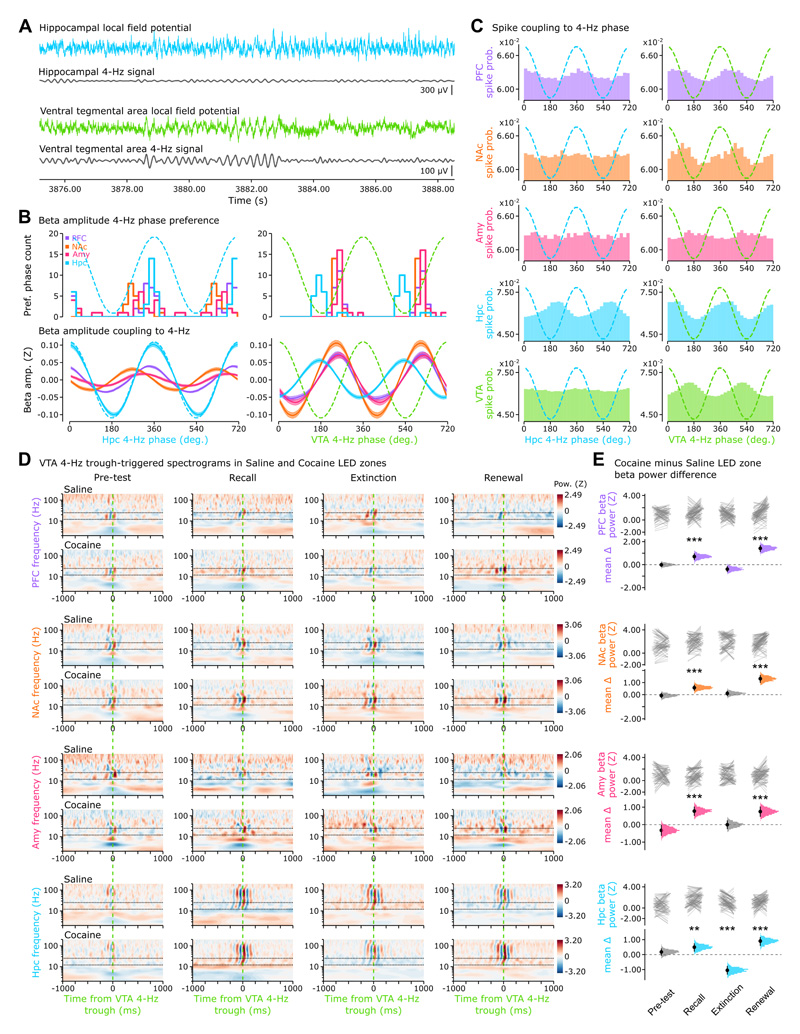
Multiregional beta activities are coupled to VTA 4-Hz oscillations and stronger during memory retrieval. **(A)** Example LFP trace with its 4-Hz signal in Hpc and VTA. **(B)** Preferred phase (*top*) and amplitude (*bottom*) of PFC, NAc, Amy and Hpc beta-band activities relative to Hpc (*left*) and VTA (*right*) 4-Hz phase (dashed lines; two 4-Hz cycles for clarity). Bottom: each color-coded thick line and its filled error represent mean ±SEM for one region. **(C)** Mean spike probability of PFC, NAc, Amy, Hpc and VTA neurons as a function of Hpc (left) and VTA (right) 4-Hz phase (dashed lines; two cycles for clarity; PFC n=421, NAc n=292, Amy n=247, Hpc n=477, VTA n=132 cells). **(D)** Power spectrograms of the PFC, NAc, Amy and CA1 Hpc LFPs during cocaine-paired versus saline-paired LED activations across tests, relative to VTA 4-Hz troughs. For each spectrogram, the two horizontal dashed black lines represent the beta-frequency (15-25 Hz) range; the vertical dashed green line represents the reference VTA 4-Hz troughs. **(E)** Corresponding changes in VTA 4-Hz trough-triggered beta-band power between cocaine-paired LED minus saline-paired LED activations over tests. Top: each gray line represents the change in power for a single frequency (1-Hz increments) within the beta range when the mouse was active (speed>2cm/s) relative to saline- (left) versus cocaine-paired (right) LEDs. Bottom: corresponding cocaine minus saline mean difference estimation distribution; *black-dot*: mean difference estimate; *filled-curve*: distribution of mean difference estimates; *black-lines*: 95% confidence interval. Note that compared to the saline-paired LED, the VTA 4-Hz trough-triggered beta-band signals associated with cocaine-paired LED activation are stronger in recall and renewal. *** p < 0.001; ** p < 0.01, permutation test, with Bonferroni correction for multiple comparisons; n = 4 stages.

We thus assessed how beta-band activities detected in individual regions are collectively orchestrated. Large-scale synchrony between multiple transient activities can be reported by slower oscillations ^34,50–52,57,64–66^. Notably, cross-regional phase-amplitude coupling by 4-Hz oscillations synchronizes faster (gamma-band) activities across hippocampal CA1, PFC, and VTA during spatial working memory ^67^. Here, we extracted 4-Hz signals from Hpc and VTA LFPs (Figures 4A and S2B). We thus evaluated the phase modulation exerted by these 4-Hz signals on each beta amplitude signal. PFC, NAc, and Amy betas were more strongly phase-modulated by VTA than Hpc 4-Hz (Figure 4B; p < 0.001, paired permutation test for VTA-minus-Hpc 4-Hz phase modulation scores, with Bonferroni correction for multiple comparisons, n = 4 regions). The amplitude of these beta oscillations shared the same phase preference for VTA, but not Hpc, 4-Hz (Figure 4B). While more strongly modulated by local 4-Hz, Hpc beta was still well-modulated by VTA 4-Hz; exhibiting an earlier phase preference compared to that shared by PFC, NAc and Amy beta signals (Figure 4B). In fact, the region-pairwise difference between the VTA 4-Hz preferred phases of PFC, NAc, Amy, and Hpc betas decreased across conditioning days (Figure S5A-C), a phase alignment indicating synchronization of brain-distributed activities. VTA 4-Hz exerted preferential modulation of beta over the other faster (gamma) signals, which was not the case for Hpc 4-Hz (Figure S5D-G). Moreover, cross-regional spiking tended to be more modulated by VTA than Hpc 4-Hz (Figure 4C). Beta power relative to VTA 4-Hz troughs was also stronger for cocaine- than saline-paired LED activations during recall compared to pre-test; a contrast that disappeared in extinction but re-emerged during renewal (Figure 4D,E). A similar effect of VTA 4-Hz trough-related beta-band power occurred with another drug (morphine) in our place-LED task (Figure S6A,B), indicating that the identified pattern of beta coordination is not unique to cocaine. Interestingly, we observed a similar but weaker activity profile in the cocaine-conditioned place preference task (Figure S6C-F), suggesting that the described cross-network pattern of transient beta-band coordination is linked to the discrete sensory cues controlled by animal’s behavior in our place-LED task. Altogether, these results relate VTA 4-Hz with the large-scale orchestration of brain-distributed beta-band activities.

### VTA 4Hz-paced glutamatergic cell spiking reflects multiregional beta coordination

**Figure 5:**
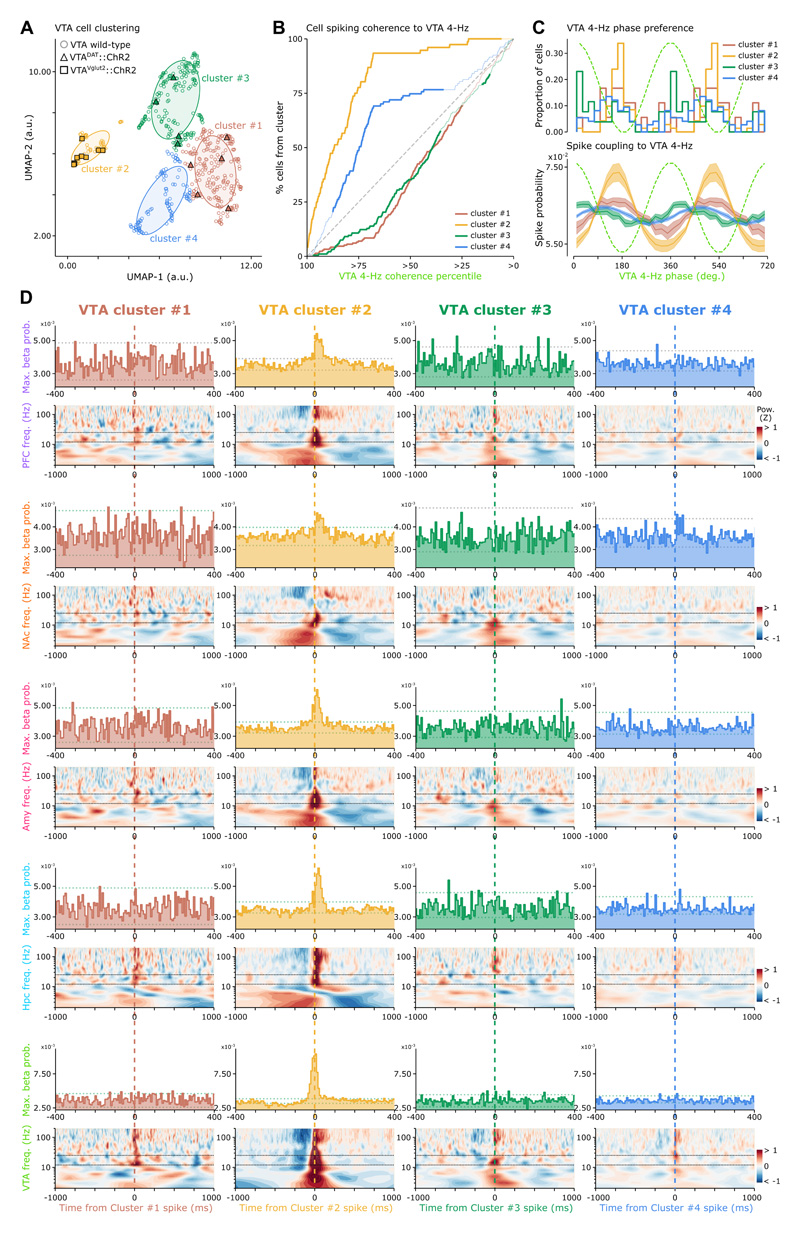
VTA glutamatergic cells are tuned to local 4-Hz and distributed beta-band signals. **(A)** VTA neuron electrophysiological clustering using dimensionality reduction (UMAP) on spike waveforms. This was applied to VTA neurons recorded in wild-type mice (each ○ represents one neuron), and those recorded and optogenetically identified (ChR2-optotagged) as dopaminergic (Δ) versus glutamatergic (□) cells in DAT-Cre versus Vglut2-Cre mice, respectively (see Figure S7A-H). Note that optotagged glutamatergic VTA^Vglut2^::ChR2-eYFP neurons fall into cluster #2. **(B,C)** Putative VTA glutamatergic neurons are most strongly coupled to VTA 4-Hz. **(B)** Comparing cluster population spike coherences to VTA 4-Hz. For each VTA cell, we calculated its spike-phase coherence to VTA 4-Hz and obtained, for each cluster, the percentage of cells (y-axis) with spike-phase coherence above a given population percentile (x-axis). Thick lines show significant over- or under-representation for that color-coded VTA cluster. Note that ~three quarters of the putative VTA glutamatergic population (cluster #2) are in the top quartile of VTA 4-Hz coherence-ranked cells. **(C)** Similarly, putative VTA glutamatergic cells (cluster #2) are most phase-locked to 4-Hz phase, with maximum spike probability towards the end of the descending slope. For each cluster, top: preferred VTA 4-Hz phase preference; bottom: firing probability as a function of VTA 4-Hz phase (thick line: group mean; shaded error: ± SEM). **(D)** Spectral responses of individual regions (rows) relative to neuronal spiking from each VTA cluster (columns). Top: cell-averaged probability distributions of time of maximum beta power relative to spike times. Horizontal dotted lines: 95% confidence interval of null distribution. Bottom: average (speed-controlled subtracted) LFP spectrogram responses to spike times. For each cell, a speed-matched control spectrogram was subtracted from its corresponding spike-averaged raw spectrogram (Figure S7I). For each panel, the two horizontal dashed black lines represent the beta-frequency range. Note that putative VTA glutamatergic cell spiking (cluster #2) precedes a strong increase in cross-regional beta power.

We thus explored the cellular substrates that would allow VTA to engage in cross-network beta coordination. The VTA hosts diverse neuronal populations where dopaminergic and glutamatergic cells both influence downstream regions ^37,40,48,49,68,69^. To start assessing whether a particular VTA population supports cross-network coordination, we used the spike waveforms of individual VTA neurons as feature data to perform unsupervised cell clustering (Figure 5A). Extracellular recordings alone do not allow directly identifying molecularly-defined cell types. We thus also considered the spike waveforms of neurons recorded from the VTA of dopamine transporter (DAT)-Cre and vesicular-glutamate transporter-2 (Vglut2)-Cre mouse lines, further optogenetically identified (optotagged) as dopaminergic and glutamatergic cells, respectively (Figure S7A-H). VTA optotagged Vglut2 cells fell into one neuronal cluster, which we inferred as the putative glutamatergic population (Figure 5A; cluster #2). VTA optotagged DAT cells spanned two putative dopaminergic clusters (Figure 5A; clusters #1 and #3).

Leveraging this neuronal clustering, we observed that the putative glutamatergic VTA population showed the strongest spike-phase coherence to VTA 4-Hz (Figure 5B), exhibiting highest firing probability just before 4-Hz troughs (Figure 5C). Using the LFPs of each region, we then computed the spectral response to the spike times of each VTA population (controlling for animal’s speed). Natural VTA glutamatergic spiking preceded transient increases of beta-band power in PFC, NAc, Amy, and Hpc (Figures 5D and S7I; cluster #2). VTA glutamatergic neurons, paced at 4-Hz, could thus play a central role in the coordination of brain-distributed beta-band activities.

### Stimulation of VTA glutamatergic cells drives multiregional beta coordination and orientates exploratory behavior

**Figure 6:**
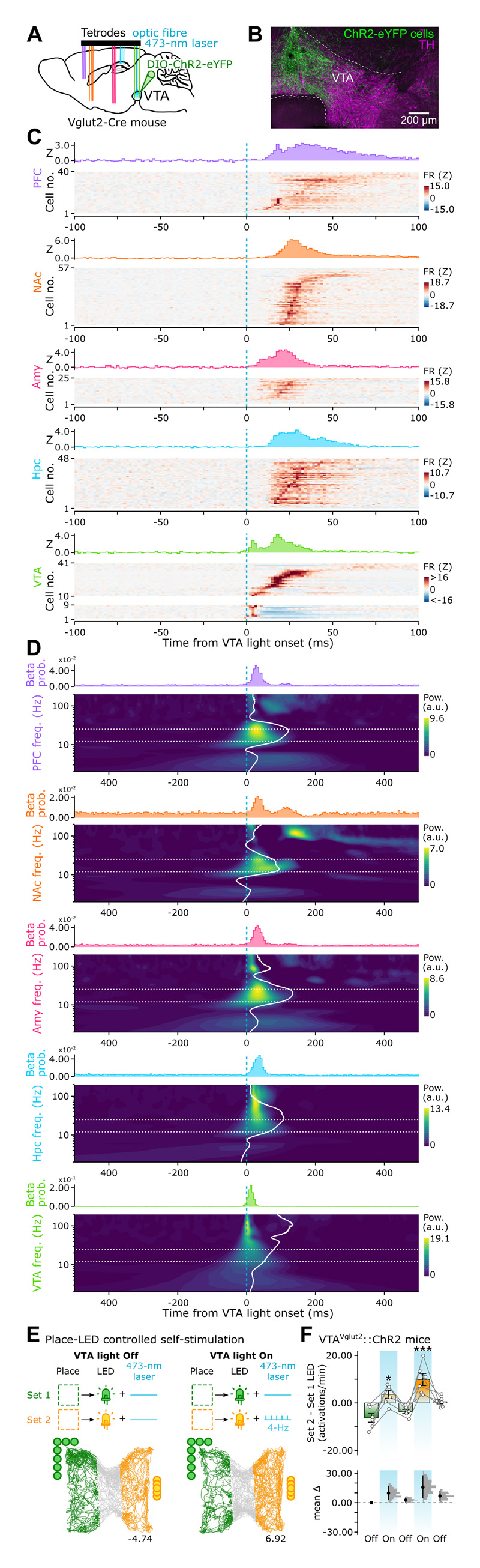
Activating VTA glutamatergic neurons entrains cross-regional activities and orientates motivated behavior. **(A-B)** Quintuple-brain-region recording with bilateral optic fibers **(A**) targeting VTA Vglut2 glutamatergic neurons transduced with ChR2 **(B**, VTA coronal section with tyrosine hydroxylase staining). Note transduction is biased towards medial VTA. **(C)** VTA Vglut2::ChR2 neuron stimulation entrains neuronal firing across regions. For each region: the histogram (top) shows the mean z-scored population firing response relative to VTA light onset; the raster (bottom) shows the corresponding individual responses (one cell per row). For VTA cells, the activity raster is split into two subpopulations, with the bottom plot showing ChR2-optotagged neurons with fast (<5-ms) response to light. Only significantly modulated cells are shown. **(D)** Concomitantly, VTA Vglut2::ChR2 neuron activation increases cross-regional beta power. Top: animal-averaged probability distributions of time of maximum beta power relative to light onset. Bottom: animal-averaged spectrogram response to light onset; vertical white solid trace: average spectral signature; horizontal white dotted lines: beta-band. **(E-F)** Place-LED controlled stimulation of VTA Vglut2::ChR2 neurons biases behavioral preference. **(E**) Top: experiment design. Mice explored the test enclosure equipped with two place-LED controllers (10-min sessions). The first (VTA light-Off) session allowed identifying the initial preference of each mouse for one place-LED set. In subsequent light-On sessions, the place-selective activation of the initially non-preferred LED resulted in 4Hz-patterned light pulse delivery to VTA Vglut2::ChR2 neurons. These sessions alternated with additional light-Off sessions (laser inactivated). Bottom: Example animal paths in VTA light-Off (left) and light-On (right) sessions. Colored lines show animal path segments in LED trigger zones. **(F**) Quantification of place-LED preference using a difference estimation plot. Top: raw data points, with each group of five connected points reporting the difference between the initially non-preferred (paired with VTA 4-Hz photo-stimulation) place-LED (Set 2) minus the preferred (unpaired) place-LED (Set 1) activation for one mouse across sessions; bar charts: average (mean±SEM) preference. Lower panel: corresponding effect size, using the mean difference for each session compared to the initial light-Off-session. *Black-dot*, mean difference; *filled-curve:* distribution of mean differences; *black-lines*, 95% confidence interval; *** p<0.001; and * p<0.05; repeated ANOVA for pairwise session-session interactions; versus initial light-Off session, with Bonferroni correction for multiple comparisons.

To test this, we assessed the cross-network-level effects of VTA glutamatergic neuron activation. We transduced the VTA of Vglut2-Cre mice with the blue light (473 nm)-driven activator channelrhodopsin-2 (ChR2). In these VTA^Vglut2^::ChR2 mice, we performed parallel PFC, NAc, Amy, Hpc, and VTA ensemble recordings with brief VTA light-delivery (Figure 6A,B; 20-ms pulses). Remarkably, activating VTA Vglut2::ChR2 cells entrained neuronal spiking in all regions (Figure 6C). This occurred with a noticeable latency (PFC, 32.4; NAc, 29.2; Amy, 22.8; Hpc, 27.6 ms; median latency of spiking response), which suggested local computation of incoming inputs ^70^. This remote spike entrainment occurred with transiently enhanced beta-band power (Figures 6D and S7J; median latency of beta power peak: PFC, 26.4; NAc, 38.4; Amy, 31.2; Hpc, 32.8 ms). These brain-distributed spiking and spectral responses indicated that VTA glutamatergic cells could constitute a diverging pathway for the actuation of cross-network beta-band coordination.

We further evaluated the behavioral effect of VTA glutamatergic cell activation, using 4Hz-patterned optogenetic stimulation directly controlled by animal’s location in the test enclosure (Figure 6E,F). For this, we identified the initial preference of each mouse for one place-LED set, before any VTA 473-nm light delivery. Mice next continued to explore the enclosure across additional sessions. In “VTA light On” sessions, they could trigger 4Hz-patterned stimulation of VTA Vglut2::ChR2 neurons by selectively entering the zone paired with their initially non-preferred LED (Figure 6E). These sessions alternated with “VTA light Off” sessions with no stimulation (laser inactivated). Remarkably, in sessions with 4Hz-self-stimulation, mice switched their preference to the associated place-LED (Figure 6F). This showed that mimicking 4Hz activity of VTA glutamatergic neurons is sufficient to reproduce the cocaine-biased behavior marking the recall and renewal stages of our conditioned cue-place preference task.

### VTA 4-Hz phase-informed suppression of cross-network beta coordination prevents renewal of cocaine-paired memory

**Figure 7:**
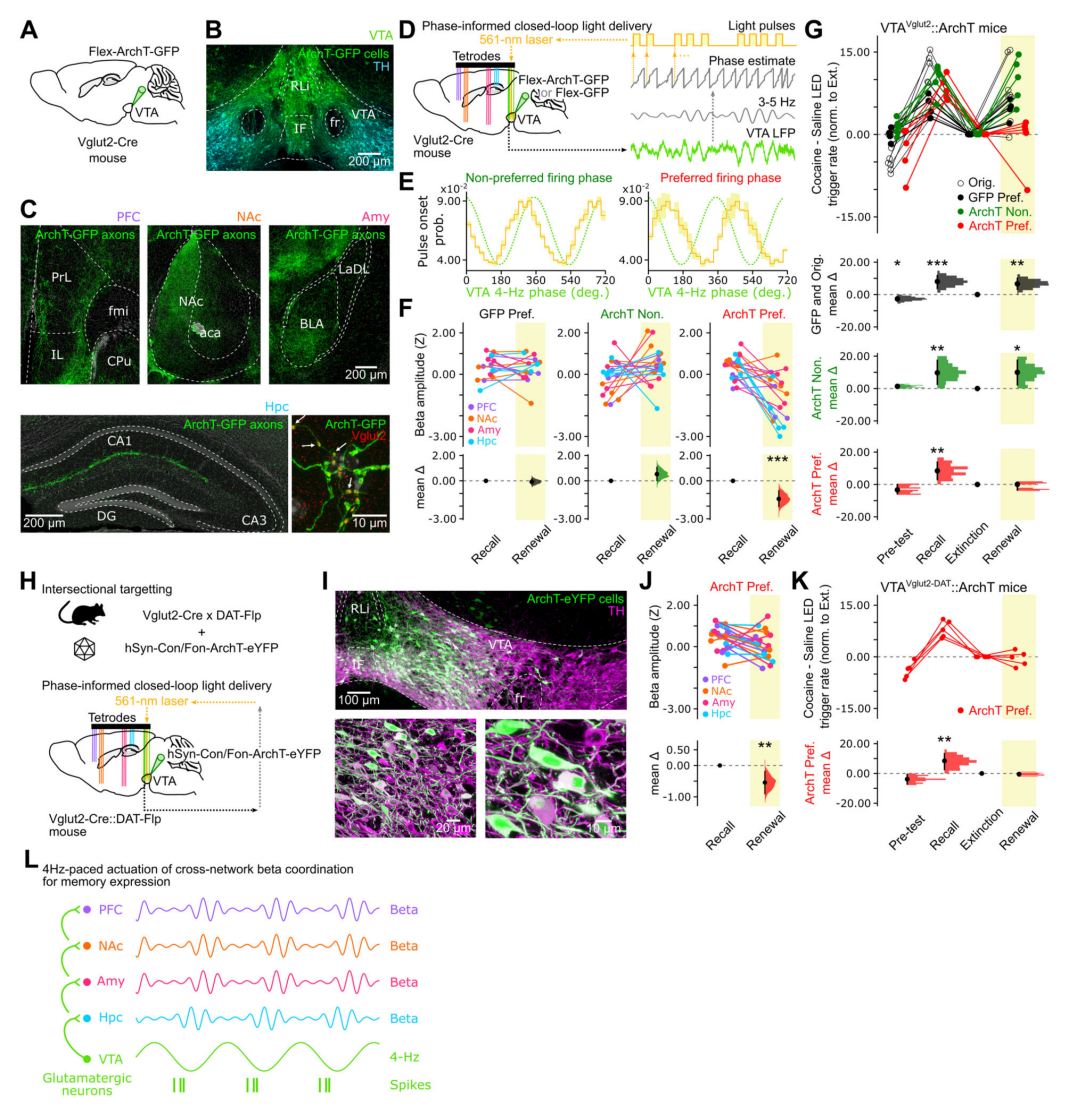
Phase-informed silencing of VTA glutamatergic neurons prevents renewal of cocaine-paired memory. **(A-C)** VTA Vglut2 diverging pathway. VTA of Vglut2-Cre mice transduced with ArchT-GFP **(A)**. ArchT-GFP expression in medial VTA neurons **(B**; with tyrosine hydroxylase staining) and their projections in PFC, NAc, Amy and Hpc **(C)**. DAPI-stained nuclei. Bottom-right: ArchT and Vglut2 co-expressing axon terminals in dorsal CA1. **(D)** Quintuple-brain-region recording with VTA 4-Hz phase-dependent bilateral VTA yellow-light delivery targeting glutamatergic neurons expressing either ArchT-GFP or GFP-only. **(E)** Laser activation as a function of VTA 4-Hz phase. Light (125-ms) pulse delivered with highest probability at the non-preferred versus the preferred phase subspace of VTA glutamatergic neurons (Figure 5C). **(F,G)** Changes in beta power and cocaine-LED oriented behavior. **(F)** In ArchT-GFP mice, inhibiting VTA glutamatergic cells during renewal reduced PFC, NAc, Amy and Hpc beta amplitude for preferred (ArchT Pref.), but not non-preferred (ArchT Non.), 4-Hz phase light-delivery. This did not happen in preferred-phase GFP-only mice (GFP Pref.). For each downstream region: *upper panel*, pairs of connected data points reporting the beta amplitude during renewal (laser-powered) compared to recall (laser-not-powered); *lower panel*, effect size for changes in beta power during renewal versus recall. *** p<0.001, paired permutation test. **(G)** This intervention prevented renewal of cocaine-LED oriented behavior in ArchT-GFP mice when performed at the preferred 4-Hz phase (ArchT Pref.). Upper panel: distribution of individual data points, with each set of four connected points reporting the difference in cocaine-paired minus saline-paired LED activations for one mouse across tests, compared to extinction (*black-filled/-unfilled:* GFP-only/wild-type mice (Figure 1G); *red/green:* ArchT-GFP; ** p<0.01; * p<0.05; repeated ANOVA versus Extinction, with Bonferroni correction for multiple comparisons). Lower panel: effect size with paired mean difference for each test compared to extinction; *black-dot*, mean difference; *filled-curve*: distribution of mean differences; *black-lines*: 95% confidence interval. **(H-K)** VTA Vglut2;DAT neuron suppression reduced downstream beta amplitude and prevented renewal of cocaine-biased behavior. **(H)** Intersectional strategy for VTA glutamatergic/dopaminergic neuron targeting in double-transgenic Vglut2-Cre;DAT-Flp mice injected with a Cre- and Flp-dependent ArchT-eYFP construct (top), used with quintuple-brain-region recordings and phase-informed bilateral VTA light delivery (bottom). **(I)** VTA coronal section (at different magnification) showing neuronal co-expression of ArchT-eYFP (green) and TH (magenta). This intervention reduced downstream beta power **(J)** and blocked cocaine-LED oriented behavior **(K)** in renewal. ** p < 0.01, paired permutation test **(J)** and repeated ANOVA versus Extinction, with Bonferroni correction for multiple comparisons **(K**). **(L)** Summary schematic. Retrieving a learnt (drug-cue-place) association involves transient coordination of multiple short-lived beta-paced network activities that are co-enhanced in recall, decrease in extinction and re-occur in renewal. This brain-distributed pattern is actuated by 4Hz-paced VTA glutamatergic cells forming a one-to-many-region pathway. This cross-network coordination could yield a cohesive (meta) ensemble of neurons serving robust memory expression.

To determine whether glutamatergic VTA projections form a divergent pathway exerting a cross-brain-network influence, we transduced VTA Vglut2 neurons with the yellow light (561 nm)-driven silencer Archaerhodopsin-T (ArchT; Figure 7A,B). In these VTA^Vglut2^::ArchT mice, Vglut2-expressing VTA axonal projections innervated many regions including those recorded here (Figure 7C). Retrograde viral vector-mediated tract tracing confirmed that the VTA Vglut2 neurons projecting to one region (e.g., the Hpc) can also project to the others (Figure S7K-Q). These findings showed that VTA glutamatergic neurons instantiate a diverging (one-to-many-region) pathway.

To directly evaluate the role of this pathway in beta coordination during memory renewal, we deployed closed-loop optogenetic silencing of VTA Vglut2 neurons according to real-time tracking of VTA 4-Hz phase (Figure 7D). We applied this to both VTA^Vglut2^::ArchT mice and control VTA^Vglut2^::GFP mice (where VTA glutamatergic neurons expressed the GFP-only construct); monitoring PFC, NAc, Amy, Hpc, and VTA activity combined with VTA light delivery at either the preferred or the non-preferred (opposite) 4-Hz phase subspace of VTA glutamatergic neuron firing (Figure 5C) during our task (Figure 7E-G). By operating this feedback during the recall stage (without light delivery), we confirmed real-time detection of the highest versus the lowest VTA 4-Hz phase preference of VTA glutamatergic neurons (Figure 7E). Closed-loop intervention with actual VTA 561-nm light delivery during renewal significantly suppressed beta amplitudes in downstream (PFC, NAc, Amy and Hpc) regions when applied at the preferred, but not the non-preferred, 4-Hz phase (Figure 7F). Critically, preventing beta-band coordination by closed-loop suppression of VTA glutamatergic cells selectively at their preferred 4-Hz firing phase prevented renewal of cocaine-biased behavior in VTA^Vglut2^::ArchT mice (Figure 7G). Closed-loop VTA light delivery at the preferred 4-Hz phase in VTA^Vglut2^::GFP control mice did not prevent the re-emergence of cocaine-biased behavior (Figure 7G). These results showed that suppressing PFC–NAc–Amy–Hpc beta coordination by 4-Hz phase-informed silencing of the VTA glutamatergic population prevented post-extinction renewal of cocaine-biased behavior.

A large number of studies have implicated dopamine in memory (e.g., ^38,39,46,71–73^). Our results showed that cross-network beta-band activity reports cocaine-paired memory recall and renewal. Yet, by computing the LFP spectrograms of individual regions, we could not relate VTA dopamine population spiking to clear changes in beta-band power (Figure 5D). Our findings nevertheless indicated that the activity of some dopamine neurons is coupled to VTA 4-Hz (Figure 5C), which phase relates to beta signal modulation (Figure 4). VTA dopamine neurons constitute a heterogeneous population, with some of its members co-expressing glutamate ^48,74–78^. We thus used an intersectional targeting strategy to manipulate the subset of genetically-defined VTA glutamate neurons that are also dopaminergic (Figure 7H). We transduced the VTA of double-transgenic Vglut2-Cre;DAT-Flp mice with a viral construct for ArchT-eYFP expression conditional to the presence of both Cre and Flp recombinases (Figure 7I). We used these VTA^Vglut2-DAT^::ArchT mice with quintuple-brain-site recordings and 4-Hz phase-locked light delivery during renewal, as before. Closed-loop suppression of VTA Vglut2-DAT neurons reduced the power of downstream beta-band signals in VTA^Vglut2-DAT^::ArchT mice (Figure 7J), by ~37.6 % of that in VTA^Vglut2^::ArchT mice (Figure 7F), along with preventing renewal of cocaine-biased behavior (Figure 7K). This showed that 4Hz-paced VTA glutamate-dopamine neuron activity is necessary in post-extinction renewal of cocaine-paired memory expression.

## Discussion

In this study, we uncover a neural pattern of brain network activities that is actuated by a VTA diverging pathway and reports the initial recall and post-extinction renewal of cocaine-paired memory. This pattern couples within a short (beta-band) timescale a set of brain-distributed regions using 4-Hz-paced VTA glutamatergic neurons, which closed-loop suppression prevents the re-emergence of cocaine-biased behavior.

### Cross-network coordination in retrieval of learned drug-cue-place association

The renewal of cocaine-biased actions highlights the persistent nature of drug memories. This robustness could reflect the distributed nature of this type of memory, engaging neuronal populations from not one but multiple brain regions. Accordingly, we modelled cocaine-biased behavior, from initial recall to extinction to renewal, while recording network oscillations and neuronal ensembles from five regions (Figure 1). This selection was informed by previous work that dissected region- and pathway-specific contributions to separable components of cocaine-paired memory. For example, pharmacological manipulations have implicated Amy ^22^ and NAc ^79^ in cue-elicited drug seeking; and PFC ^80,81^ and Hpc ^60,82,83^ in context-dependent modulation of drug-paired memory retrieval. Numerous studies have implicated VTA in various aspects of drug-seeking behavior ^15,42,84^. Combining behavioral assays of drug-place conditioning with Hpc (and downstream NAc) recordings has recently begun to describe how drugs of abuse alter internal dynamics relevant to drug-associated memory ^27,28,85^. Our findings build upon these studies by revealing that the neuronal activities localized in each of these regions are organized into a coherent (meta-) ensemble, whose tight temporal structure serves memory expression.

By investigating higher-order patterns of coordination, our findings show that the structure of neural activity during recall and renewal of cocaine-paired memory resonates in the beta-frequency range (Figure 2). This could report various transient operations associated with goal-oriented behavior, including the processing of a global reward-seeking signal, the retrieval of learnt drug-cue association, or the translation to a motivated behavior readout. By training machine learning models to identify the active place-LED set from population spiking vectors, we show that neurons representing task-relevant information are more coupled to beta signals (Figure 3). Neuronal spiking is modulated with reference to beta-frequency signals in either PFC, NAc, Amy, or Hpc CA1; in line with the observation that these signals are tightly coordinated. This beta-band coordination is consistent with theoretical work suggesting that neural synchrony over longer conduction delays (i.e., between distally connected regions) is more readily supported by the beta- (rather than gamma-) timescale ^86^. Our observation that cocaine-paired memory implicates cross-network beta coordination is also in line with the hypothesis that enhanced beta activity can report a cognitive state detrimental to flexible behavior ^87^. On the (brain) spatial scale, this preference for beta (fast yet slower than gamma) coordination suggests the recruitment of larger neuronal ensembles ^52,64,66^. This could exert greater influence on animal’s behavior, biasing it towards salience-associated stimuli. In line with this, past work showed that neuronal responses to reward-predicting stimuli increases with reward magnitude^88,89^. The cross-network pattern identified here might thus report an “incentive salience” signature ^2,90,91^. Together, these findings support the view that the robustness of cocaine-paired memory expression relates to the temporally-patterned coordination of distributed networks into a cohesive (beta-tuned) spiking structure. Via such distributed yet coordinated activities, downstream neurons would receive multiple inputs converging at a timescale that is fast enough to provide an effective neural syntax for memory and its translation into a behavioral output ^50,57,92–94^. Accordingly, identifying means to disrupt this cross-network organization would allow preventing renewal of drug-biased behavior.

### A neural substrate of cross-regional communication

Cross-region communication is more likely facilitated by slower frequencies, which are coherent across brain structures and report rhythmic fluctuations of large-scale neural activity ^34,55,57,64,95^. Notably, 4-Hz phase modulation of gamma activities in PFC, Hpc and VTA is enhanced during working memory demand ^67^. Our data-driven method extracted 4-Hz signals in VTA and Hpc (Figure 4). We observed strong VTA 4-Hz phase modulation of beta signal amplitude in PFC, NAc, Amy and Hpc – which was also selective for beta over gamma signals in PFC, NAc and Amy (Figure S5D-G). We also observed tighter preferred-phase alignment of PFC, NAc and Amy beta signals for VTA compared to Hpc 4-Hz phase (Figure 4B). Moreover, VTA population firing increases prior to strong instances of beta-bout activity in the other regions (Figure 3G) and shows 4-Hz modulation when individual VTA cell responses are triggered by beta troughs (Figure 3H). These results suggested that VTA plays a leading role in the transient organization of cross-network activities during memory retrieval. This is consistent with work relating VTA to reward prediction signals ^96–98^. VTA also co-targets a large array of brain regions ^99^, including those recorded here. Notably, single-cell axon tracing revealed that individual VTA neurons can project to both cortical and limbic structures ^100^. These observations indicated the existence of a VTA diverging pathway actuating cross-regional coordination of short-lived beta activities with the phase of a slow (4-Hz) rhythm.

Identifying the cellular substrate of this network coordination remained to elucidate. The VTA hosts diverse populations of molecularly defined neurons that can be distinguished by their spike waveforms ^36,67,72,101–104^. Accordingly, we leveraged an optogenetically-validated VTA cell classification suggesting the glutamatergic population plays a central role in the actuation of multiregional beta coordination (Figure 5). Putative VTA glutamatergic neurons are most strongly coupled to local 4-Hz, with preferred firing toward the end of the descending phase (Figure 5C), just prior to the 4-Hz phase with highest beta amplitude (Figure 4B). Following natural spiking of VTA glutamatergic neurons, beta power markedly increased in PFC, NAc, Amy and Hpc (Figure 5D). These observations could explain the phase alignment of cross-regional beta amplitudes to VTA 4-Hz (Figure 4B). Moreover, stimulating VTA Vglut2 neurons entrained neuronal spiking in all recorded regions and increased beta power (Figure 6A-D). Prior work showed that VTA Vglut2 cells can shape goal-oriented behavior ^37,40,105–107^. Activating VTA Vglut2 is indeed behaviorally potent: 4-Hz patterned self-stimulation of VTA Vglut2 neurons was sufficient to orientate mice towards the associated place-LED set (Figure 6E,F), mimicking the behavioral bias observed during the recall and renewal of our task. Prior studies reported that mice prefer VTA Vglut2 stimulation at a higher (40 Hz) frequency ^40,107^. While this behavioral effect was not related to natural *in vivo* electrophysiological monitoring of VTA Vglut2 neurons, the reported preference for faster frequencies could reflect an inherently rewarding effect of VTA Vglut2 stimulation ^40,105,108^; rather than the actuation of a large-scale oscillatory substrate for mnemonic retrieval of rewarding experiences. Future studies could investigate these facets of reward-paired memory retrieval, their inherent relationships, and how they may be supported across electrophysiological, molecular, and anatomical domains. Interestingly, the VTA 4-Hz phase preference of the Hpc beta signal amplitude occurred earlier than that of PFC, NAc and Amy beta signals (Figure 4B). This suggests that, while VTA 4-Hz oscillations orchestrate multiregional beta coordination, part of this induction could be encouraged by hippocampal beta activity; perhaps broadcasting spatio-contextual information to downstream reader neurons. This may explain the tendency for stronger hippocampal beta signals in the recall and renewal stages of the conditioned place preference task (Figure S6F). In the CPP task, animals are not exerting direct control over the environmental cues. This difference between the two behavioural paradigms could influence the degree of reliance on short-lived binding of brain-distributed activities.

VTA Vglut2 cells form diverging projections (Figures 7A-C and S7J-P) ^77,100^. We thus hypothesized that a one-to-many-region VTA glutamatergic pathway actuates the large-scale coordination of beta activities during memory expression. We found that VTA 4-Hz phase-triggered silencing of VTA glutamatergic neurons suppressed beta signals in PFC, NAc, Amy and Hpc and prevented re-emergence of cocaine-biased behavior (Figure 7D-G). This outcome was obtained when silencing VTA Vglut2 neurons specifically at their preferred 4-Hz phase. This supports a 4Hz-orchestrated, brain-distributed network mechanism of robust memory retrieval actuated by the VTA glutamatergic population. Interestingly, we noted beta-band correlates of VTA putative dopaminergic population spiking (Figure 5D; VTA clusters #1 and #3), albeit weaker than that of VTA putative glutamatergic population (Figure 5D; VTA cluster #2). Numerous studies have implicated dopamine in drug experience and memory; and an increasing number of studies shows that VTA dopamine neurons can co-express multiple neurotransmitters ^48,49,74–78,99,109–111^. By targeting the subset of VTA glutamate neurons expressing dopamine with 4-Hz phase-locked silencing, we found that the VTA glutamate-dopamine neuron subpopulation is required for memory renewal (Figure 7H-K). Previous work showed that VTA dopamine cells receive local inputs from VTA glutamate cells ^105,108^. Our VTA ensemble recordings also showed that stimulation of VTA glutamate neurons entrains spiking activity of other local neurons (Figure 6C), many of which are likely to be dopaminergic ^108^. The reward-predicting properties of VTA dopamine neurons are well-established ^36,39,41,98,112–115^, with their firing activity being sustained on omission of expected reward following cocaine experience ^42^. These findings indicate that while the VTA glutamatergic population coordinates cross-regional targets during memory retrieval, they could further define a window wherein short-timescale coordination of multiple networks is influenced by concomitant dopamine release. Theoretical work has predicted that glutamatergic inputs arriving from remote upstream circuits preferentially synchronize over the beta time scale ^86^. This timescale would permit the recruitment of distributed neuronal ensembles whose large spatial scale could be promoted by dopamine ^116,117^.

### Limitations of the Study

Our study identifies a brain-distributed pattern of short-lived beta-band activities that is actuated by VTA glutamatergic neurons and paced by a slower (4-Hz) oscillation. Our findings raise the exciting prospect that VTA neurotransmitter co-release at multiple temporally coordinated targets would provide fine control of transient cross-network ensemble activity, and thus the complexity of moment-by-moment internal processing of mnemonic information. Yet, the precise relationships between VTA dopamine/glutamate release dynamics, cross-regional oscillatory coordination, internal processing of learned associations and behavioral action to gain reward/drug remain to be elucidated. Future work aiming to delineate these relationships would require novel methodological approaches. Further, we considered five regions, but more are engaged by drug experience and memory. Whether additional networks participate in the described pattern of VTA 4Hz-paced beta-band coordination remains to be assessed. This could leverage higher-density recordings. Additional regions would be expected to receive VTA glutamatergic projections. This study involved adult male mice able to carry a multiregional electrode drive for brain-distributed ensemble recordings. Whether the described pattern of cross-network coordination is generalizable to adult female mice and young mice remains to assess. By focusing on drug experience, our study supports the idea that binding together distinct networks in this temporally-structured manner may constitute an organizational principle of robust memory expression. The extent to which this applies to other memories remains unevaluated. Moreover, drug-related memories can precipitate the transition towards loss of behavioral control and addiction. Our paradigm was confined to ~1 week. Monitoring brain networks over a much longer period in animals self-administering a drug would provide important insights into the neural dynamics of addiction.

## STAR Methods

### Resource Availability

#### Lead Contact

Further information and requests for resources and reagents should be directed to and will be fulfilled by the Lead Contact, David Dupret (david.dupret@bndu.ox.ac.uk).

#### Materials Availability

This study generated viral constructs for the Cre-dependent and Flp-dependent expression of ArchT-eYFP in small quantities and can be requested from david.dupret@bndu.ox.ac.uk while our stocks last, with priority to replication projects, and those entering into collaboration with us.

### Experimental Model and Study Participant Details

#### Animals

These experiments used adult (4–6 months old) male C57BL/6J wild-type mice (Charles River Laboratories, UK) and transgenic mice heterozygous for the transgene expressing the Cre recombinase under the control of either the vesicular-glutamate transporter-2 (Vglut2) or the dopamine transporter (DAT) promoter (Jackson Laboratories; obtained from C57BL/6J crossed with Vglut2-ires-cre B6J.129S6(FVB)-Slc17a6tm2(cre)Lowl/MwarJ, stock number 028863, RRID: IMSR_JAX:028863; or with DAT-ires-cre B6.SJL-Slc6a3tm1.1(cre)Bkmn/J, stock number 006660, RRID: IMSR_JAX:006660) or for the transgene expressing the optimized Flp recombinase under the control of the DAT promoter (Jackson Laboratories; obtained from C57BL/6J crossed with DAT-P2A-Flpo Slc6a3em1(flpo)Hbat/J, stock number 035436, RRID: IMSR_JAX:035436)^120–122^. Animals were housed with their littermates up until the start of the experiment. All mice held in IVCs, with wooden chew stick, nestlets and free access to water and food *ad libitum* in a dedicated housing facility with a 12/12 h light/dark cycle (lights on at 07:00), 19–23°C ambient temperature and 40–70% humidity. Experimental procedures performed on mice in accordance with the Animals (Scientific Procedures) Act, 1986 (United Kingdom), with final ethical review by the Animals in Science Regulation Unit of the UK Home Office.

### Method Details

#### Surgical procedure

All surgical procedures were performed under deep anesthesia using isoflurane (0.5–2%) and oxygen (2 l/min), with analgesia provided before (0.1 mg/kg vetergesic) and after (5 mg/kg metacam) surgery. To generate expression of either ChR2-eYFP, ArchT-GFP or GFP-only in VTA neurons, we used a Cre-loxP approach by injecting the Cre-inducible recombinant adeno-associated viral vector rAAV2-EF1a-DIO-hChR2(H134R)-eYFP-WPRE (from K. Deisseroth at UNC Vector Core), rAAV2-CAG-FLEX-ArchT-GFP (from E.S. Boyden at UNC Vector Core), or rAAV2-CAG-FLEX-GFP (from E.S. Boyden at UNC Vector Core), respectively. Viral injections were targeted bilaterally to VTA using stereotaxic coordinates (-3.1mm anteroposterior from bregma, ±0.4 mm lateral from bregma, and -3.85 mm ventral from the brain surface; 150nl per site) at a rate of 100 nl/min using a glass micropipette lowered to the target site and held in place for 5 min after virus delivery before being withdrawn. For electrophysiological recordings, mice were implanted with a single microdrive containing all (12 or 14) independently movable tetrodes. Tetrodes were constructed by twisting together four insulated tungsten wires (12 μm diameter, California Fine Wire) which were briefly heated to bind them together into a single bundle. Each tetrode was loaded in one cannula attached to a 6 mm long M1.0 screw to enable its independent manipulation of depth. The microdrive layout was organised on the horizontal plane of the brain ^123^ so that the tetrode-holding cannulas were positioned according to the coordinates of region-centred craniotomies (see below), with individual tetrodes cut at the appropriate length so as to implant each tetrode ~300 μm above its target region on the vertical plane of the brain. The microdrive was implanted under stereotaxic control with reference to bregma. For dorsal hippocampal CA1, tetrodes were implanted by first identifying central coordinates -2.0 mm anteroposterior from bregma, +1.7 mm lateral from bregma as a reference to position each individual tetrode contained in the microdrive, initially implanting tetrodes above the pyramidal layer (-1.1 mm ventral from brain surface). A similar approach was used for tetrodes aimed at the medial prefrontal cortex using central coordinates +1.7 mm anteroposterior from bregma, +0.3 mm lateral from bregma, and an initial -1.5 mm ventral from brain surface; at the ventral tegmental area using central coordinates -3.1 mm anteroposterior from bregma, +0.4 mm lateral from bregma, and an initial -3.85 mm ventral from brain surface; at the nucleus accumbens using central coordinates +1.4 mm anteroposterior from bregma, +1.0 mm lateral from bregma, and an initial -4.0 mm ventral from brain surface; and at the amygdala using central coordinates -1.65 mm anteroposterior from bregma, +2.75 mm lateral from bregma, and an initial -3.8 mm ventral from brain surface. The distance between neighboring tetrodes inserted in each brain region was 350 μm. Following the implantation, the exposed parts of the tetrodes were covered with paraffin wax, after which the drive was secured to the skull using dental cement. For extra stability, four stainless-steel anchor screws were inserted into the skull before the drive was implanted. Two of the anchor screws, inserted above the cerebellum, were attached to 50 μm tungsten wires (California Fine Wire) and further served as a ground and reference electrodes during the recordings. For the recordings, each tetrode was lowered along the vertical axis to reach its target region, using the rotations applied to its tetrode cannula-holding screw and the spectral content of its LFPs, with final depth position subsequently confirmed by histology of anatomical tracks. For optogenetic manipulations, two optic fibers (230 μm diameter, Doric Lenses, Canada) were incorporated into the microdrive designed to bilaterally deliver light to VTA and implanted 10 days after viral injections.

To further assess whether the VTA glutamatergic neurons that project to one brain region also project to the other recorded regions, we performed retrograde viral vector-mediated tract tracing using Vglut2-cre mice injected with 50 nL of AAVrg-EF1a-DIO-FLPo-WPRE-hGHpA (from Li Zhang Addgene viral prep # 87306-AAVrg; http://n2t.net/addgene:87306 ; RRID:Addgene_87306)^124^ in dorsal CA1 hippocampus (AP -2, ML ±1.2 and AP -2.3, ML ±1.4 mm from bregma, and DV -1.2 from the pia) and AAV5-EF1a-fDIO-eYFP-WPRE (from K. Deisseroth at UNC Vector Core) in VTA-(AP -3.2, ML +.35 from bregma, and 4.02 mm from the pia under a 5° angle in the lateral to medial direction) (Figure S7J-P).

To assess whether the VTA neurons that are genetically defined to co-express glutamate and dopamine are required for cross-network beta-band signaling and renewal of cocaine-paired memory, we produced and injected a viral construct allowing the Cre-dependent and Flp-dependent expression of ArchT-eYFP in the VTA of double-transgenic Vglut2-Cre;DAT-Flp mice (Figure 7H,I). The corresponding pAAV-hSyn-Con/Fon-ArchT-eYFP construct was cloned in two stages. First, pAAV-hSyn-Coff/Fon-ArchT-eYFP has been cloned. Plasmid vectors pAAV-CamKII-ArchT-GFP (a gift from Edward Boyden, Addgene plasmid #37807; http://n2t.net/addgene:37807; RRID: Addgene_37807)^125^, pAAV-hSyn-Coff/Fon-hChR2(H134R)-eYFP (a gift from Karl Deisseroth, Addgene plasmid #55648; http://n2t.net/addgene:55648; RRID: Addgene_55648)^126^ and the combination of the PCR products was used to assemble two inserts that were then subcloned into the pAAV-hSyn Coff/Fon-hChR2(H134R)-eYFP vector to substitute the corresponding hChR2-eYFP coding exons with the ArchT-eYFP ones. Primers and the template plasmid DNA for the first insert (exon 1): GTTTCTGCTAGCAACCCCGACACTTACCTTAGCCAGCAGGGCCAG, GTTTCTGAGCTCGCCACCATGGACCCCATC, plasmid#37807. The PCR product was then cloned into the plasmid #55648 using NheI and SacI recognition sites thus forming the intermediate vector. For the following subcloning of the exon 2 the three PCR products were generated with the primers and the corresponding template DNAs: GTTTCTACTAGTCCTCCTGTACTCACC, GTGAGCAAGGGCGAGGAG, plasmid #55648; CTCCTCGCCCTTGCTCACTGCTACTACCGGTCGGGG, GACTCTATTTCTCATGTGTTTAGGTGGACAGGGTGAGCATCG, plasmid #37807; CCTAAACACATGAGAAATAGAGTC, CGAAGTTATGGTACCTGTGCCCCCCC, plasmid #55648. These three products were then combined in the overlapping PCR and inserted into the intermediate vector using SpeI and KpnI cloning sites forming pAAV-hSyn-Coff/Fon-ArchT-eYFP. pAAV-hSyn-Coff/Fon-ArchT-YFP vector then was used to produce pAAV-hSyn-Con/Fon-ArchT-eYFP by inverting the sequence containing part of ArchT-eYFP exon and flanked with the SpeI and KpnI restriction enzymes. The corresponding insert was produced by the PCR with the primers GTTTCTACTAGTTGTGCCCCCCCTTTTTTTTAT and GTTTCTGGTACCCCTCCTGTACTCACCTTGCC using pAAV-hSyn-Coff/Fon-ArchT-eYFP vector as a template.

#### Five-stage conditioned cue-place preference task

Following full recovery from the surgery, each mouse was first handled in a dedicated handling cloth, connected to the recording system, and exposed to an open-field enclosure to be familiarized with the recording procedure over a period of one week prior to the start of the experiment itself. During this period, tetrodes were gradually lowered to their corresponding target region using their estimated depth location, local field potentials and neuronal spike waveforms. An electrical commutator (Imetronic, Bordeaux, France) was installed above the dimly lit behavioral apparatus to hold the recording cable and allow its full rotation, preventing its twisting while the animal explored the enclosure.

The first stage of our ‘conditioned cue-place preference’ task occurred in the test enclosure (Figure 1C-E; “pre-test”). This enclosure had a bow-tie shape (outer dimension: 46 cm width; 38 cm height) and contained two LED wall-displays placed on two opposing walls (green L-shape and orange circle-shape; Figure 1D)^61,62^. Along each wall holding an LED display, we defined two spatial zones of four equally sized, un-marked quadrants. We used this test enclosure with an open-loop system (Imetronic, Bordeaux, France) to track the animal’s position in real time and generate TTL-pulses that activate in turn the corresponding LED display for up to 1.5 s. To activate an LED again, the animal had to move into one of the neighboring zone quadrants. If the mouse moved to a neighboring quadrant while the LED was still active, this movement would briefly inactivate the LED (zone quadrant exit) before re-activating it (zone quadrant entry). Mice experienced the two sets of place-LED associations (Figure 1C) while exploring the test enclosure for two days (60 minutes on the first day exposure; 20 minutes on the second day exposure). The second exposure was used to quantify the baseline (pre-test) LED activation preference of each mouse.

The second stage of this task occurred in the conditioning enclosure (Figure 1C; “conditioning”), commencing 30 minutes after the pre-test and was repeated on two successive days. To proceed, the LED display that each mouse had preferentially activated during the pre-test was attached to one of the walls of the conditioning enclosure (square shape arena; 46 cm width; 38 cm height) and the animal was allowed to first explore that enclosure for 10 minutes with the LED inactive. The animal was then briefly removed from the conditioning enclosure to receive one intraperitoneal injection of saline solution (200 μl), and immediately re-exposed to that enclosure for 30 minutes and where we emulated the behavioral pattern of LED activation that had been directly elicited by the animal during pre-test, randomly sampling from the interquartile range of the LED-on and LED-off duration distributions observed in the pre-test (mean LED-on IQR: 42–634ms; mean LED-off IQR: 46–647ms). This procedure was repeated 4 hours after the saline conditioning session, this time attaching in the conditioning enclosure the non-preferred LED from the pre-test and injecting the animal with a 12 mg/kg cocaine hydrochloride solution (200 μl; obtained from Sigma-Aldrich). The presentation of the non-preferred (cocaine-associated) LED versus the preferred (saline-associated) LED stimulus was therefore not different during these sessions, avoiding an imbalance between the amount of time each LED stimulus was shown during conditioning. Previous studies have reported that 5 to 20 mg/kg dose of cocaine is reinforcing (e.g., ^127^) and 5 to 40 mg/kg dose of morphine is reinforcing (e.g. ^128^). In an additional group of mice, the injection of cocaine used in the conditioning stage of this cue-place preference task was replaced by a 15 mg/kg morphine sulfate solution (200 μl; obtained from Martindale Pharmaceuticals Ltd) to evaluate whether the observed cross-network pattern of VTA 4Hz-paced modulation of beta-band signals was specific to cocaine experience (Figure S6A,B).

The last three task stages occurred in the test enclosure (Figure 1C; “recall”, “extinction” and “renewal”), commencing 23 hours after the last cocaine conditioning session and being all separated by 5 minutes (whilst the floor-contexts were changed; see below). In all these stages, the test enclosure was equipped with the place-LED controller, allowing mice to activate again the two LED displays as per the pre-test. In each of these three test stages, we quantified cocaine-biased behavior of each mouse using their LED trigger preference as the difference between the cocaine-paired LED activation rate minus the saline-paired LED activation rate. Both the recall and renewal stages lasted 20 minutes, with the test enclosure equipped with the floor used during pre-test. The extinction stage consisted of 10-minute blocks during each of which the animal’s preference for the cocaine-paired versus the saline-paired LED was measured. To promote behavioral extinction of cocaine-biased actions, we replaced the floor used during the pre-test, recall and renewal stages by another floor, thereby introducing a contextual change within the test enclosure while preserving its spatial layout ^129^. Thus, during the course of this protocol each mouse encountered two floors in the test enclosure: a rough texture, light color floor and a smooth texture, dark color floor. One of these two floors was used specifically for the pre-test, recall and renewal stages; whereas the other was used for the extinction stage. The floor type assigned to the extinction context was counterbalanced across animals. To further promote behavioral extinction of cocaine-biased actions, a white noise was played during all these task stages but not during extinction. During this post-recall session, we considered that the learnt drug-cue-place association was extinguished when the animal exhibited for a minimum of 30-minutes a stable reduction in its preference for activating the cocaine-paired LED compared to the preference score measured during the recall stage and the first extinction block (mean number of extinction blocks required = 6.55; IQR = 4.00-7.00). For all analyses, the final 20-minute period (last two 10-min blocks) defined the extinction stage and was used for comparisons with the 20-minute pre-test, recall and renewal stages.

#### Conditioned place preference task

In a separate group of mice, we evaluated whether the identified pattern of transient coordination of cross-network beta signals could be generalized to the conditioned place preference (CPP) task. In this task, the animal performance is scored by the difference in time spent within the initially non-preferred (later cocaine-paired) compartment minus the time spent within the initially preferred (later saline-paired) compartment, over their sum. The CPP apparatus consisted of two compartments, the dimension of each of which was equal to that of the bow-tie shape test enclosure used in our conditioned cue-place preference task (outer dimension: 46 cm width; 38 cm height). The two compartments were connected via a bridge (8-cm length, 5-cm width), during the pre-test, recall, extinction, and renewal stages. As for the conditioned cue-place preference task, following full recovery from the surgery, each mouse was first handled in a dedicated handling cloth, connected to the recording system, and exposed to an open-field enclosure to be familiarized with the recording procedure prior to the start of the CPP experiment itself. During this period, tetrodes were gradually lowered to their corresponding target region using their estimated depth location, local field potentials and neuronal spike waveforms.

On the first CPP day, mice explored the entire apparatus for 20 min (pre-test) to determine their initial preference for one of the two compartments. Then, the bridge was removed and mice were conditioned for three days with two pairing sessions each day. Conditioning was performed with respect to the initial preference of each animal (as identified during the pre-test) for one of the two compartments ^27^. In the first session, mice received intraperitoneal injection of saline solution (200 μl), before exploring the preferred compartment for 30 min (saline-paired compartment). In the second session, 4 hours later, mice received a 12 mg/kg cocaine solution (200 μl) before exploring the non-preferred compartment for 30 min (cocaine-paired compartment). On the day after (23 h after) the last conditioning session, we replaced the bridge between the two compartments and assessed CPP by allowing mice to re-explore the entire apparatus for 20 min (recall). The extinction stage next consisted of 10-minute blocks during each of which the animal’s preference for the cocaine-paired versus the saline-paired compartment was measured, as in our conditioned cue-place preference task. To promote behavioral extinction of CPP behavior, we changed the floor used during the pre-test, recall and renewal stages by another floor while preserving its spatial layout, thereby introducing a contextual change similar to the one used within the conditioned cue-place preference task. Thus, during the course of this CPP protocol each mouse encountered two floors in each CPP compartment: a rough texture, light color floor and a smooth texture, dark color floor. One of these two floors was used specifically for the pre-test, recall and renewal stages; whereas the other was used for the extinction stage. The floor type assigned to the extinction context was also counterbalanced across animals. To further promote behavioral extinction of CPP behavior, a white noise was played during all these task stages but not during extinction. We identified the extinction stage as the last two 10-min blocks marked by a stable reduction in the animal’s preference for exploring the cocaine-paired compartment compared to the CPP score measured during the recall stage, as we did for the conditioned cue-place preference task. This final 20-minute period defined the extinction stage and was used for comparisons with the 20-minute pre-test, recall and renewal stages. We replaced the original floors and activated the white noise to conduct the renewal stage.

#### Multichannel data acquisition, position tracking and light delivery

Recordings were performed at the same time (i.e., 10.00 am for Pre-test and Saline sessions; 2.00 pm for Cocaine and Post-test sessions) for all mice. The extracellular signals from each tetrode channel were amplified, multiplexed, and digitized using a single integrated circuit located on the head of the animal (RHD2164, Intan Technologies; http://intantech.com/products_RHD2000.html; pass band 0.09 Hz to 7.60 kHz). The amplified and filtered electrophysiological signals were digitized at 20 kHz and saved to disk along with the synchronization signals (transistor-transistor logic digital pulses) reporting the animal’s position tracking, LED display activations and laser activations. The location of the animal was tracked using three differently colored LED clusters attached to the electrode casing and captured at 39 frames per second by an overhead color camera (https://github.com/kevin-allen/positrack/wiki). In addition, online location of the animal was used to trigger the corresponding LED displays in an open-loop manner, using the real time movement of the animal into the trigger quadrants (Imetronic, Bordeaux, France). This place-LED controller system was also used to activate 473-nm VTA light delivery for the 4-Hz patterned stimulation of VTA glutamatergic neurons in the VTA^Vglut2^ ::ChR2 mice (Figure 6E,F). The LFPs were down-sampled to 1250 Hz for all subsequent analyses. For optogenetic interventions, 473-nm and 561-nm diode pumped solid-state lasers (Crystal Laser, models CL473-100 and CL561-100; distributer: Laser 2000, Ringstead, UK) were used to deliver light bilaterally to the VTA (~3-5 mW) via a 2-channel rotary joint (Doric Lenses Inc.).

To test for the required contribution of VTA glutamate cells in cocaine-paired memory renewal and cross-network beta coordination, we performed closed-loop optogenetic experiments to deliver VTA 561-nm light pulses in VTA^Vglut2^::ArchT, VTA^Vglut2;DAT^::ArchT, or VTA^Vglut2^::GFP mice using dynamic tracking of ongoing VTA 4-Hz phase (Figure 7D,E,H). The real-time phase estimate was obtained using the OscillTrack algorithm (https://doi.org/10.5287/bodleian:qa9ngXrzr)^130,131^ implemented in the field-programmable gate array of the Intan Technologies interface board. In these experiments, phase detection was obtained by continuously operating on the data stream coming from an input channel containing the VTA LFPs. This input channel used as the phase reference was high-pass filtered using a 1st order digital infinite impulse response filter with a corner frequency of 0.4 Hz to remove amplifier offset and electrode drift, then down-sampled 125-fold to a rate of 160 Hz for processing. The phase estimation was operated with a loop-gain of 0.0625 at a centre frequency of 4 Hz. Stimulation was triggered with a phase lead, such that the target phase aligned with the middle of the 125-ms light pulse.

#### Spike detection and unit isolation

Spike sorting and unit isolation were performed with an automated clustering pipeline using Kilosort (https://github.com/cortex-lab/KiloSort) via the SpikeForest framework (https://github.com/flatironinstitute/spikeforest) ^132,133^. To apply KiloSort to data acquired using tetrodes, the algorithm restricted templates to channels within a given tetrode bundle, while masking all other recording channels. All sessions recorded on a given day were concatenated and cluster cut together to monitor cells throughout the experiment day. The resulting clusters were verified by the operator using cross-channel spike waveforms, auto-correlation histograms and cross-correlation histograms. Each unit used for analyses showed throughout the entire recording day stable spike waveforms, clear refractory period in their auto-correlation histogram, and absence of refractory period in its cross-correlation histograms with the other units. Principal cells and interneurons were identified by their auto-correlograms, firing rates, and spike waveforms. In total, this study includes n=2,602 neurons from quintuple-brain-site multichannel recording (PFC n=421, NAc n=292, Amy n=247, Hpc n=477, VTA n=132 for the spiking analyses presented in Figures 3-5; PFC n=260, NAc n=177, Amy n=217, Hpc n=309, VTA n=70 for the spiking analysis presented in Figure 6).

### Quantification and Statistical Analysis

#### Behavioral Factor Analysis

For each mouse, we collected a set of behavioral metrics (n=11) to represent the multivariate nature of its cocaine-biased behavior during the recall, extinction, and renewal stages of our cocaine-paired memory task (see Table S1 and Figure S1E,F). For each of these behavioral metrics, we then subtracted the corresponding baseline value measured in the pre-test stage. These pre-test adjusted metrics formed a feature matrix (test stages x behavioral metrics). Each metric column of this feature matrix was then normalized by a z-score transform such that factor analysis would not be biased towards metrics with higher numerical values. We then applied factor analysis using the scikit-learn 0.19.1 package (https://pypi.org/project/scikit-learn/).

#### LFP decomposition of individual brain regions into oscillatory components

For each mouse, we applied masked Empirical Mode Decomposition (mEMD) to break down the LFP raw trace recorded in each brain region during active exploration (training the algorithm using LFP traces from each animal corresponding to the continuous 5-minute window which had the highest proportion of locomotion speeds above 2 cm.s^-1^) into its oscillatory components, namely the Intrinsic Mode Functions (IMFs; Figure S2) using the emd 0.2.0 package (https://emd.readthedocs.io/en/stable/) ^134^. As an unsupervised sifting process, EMD successively extracts the highest frequency components of a time series until only a slow residual remains ^135^. In the mEMD approach, a masking signal is added at each sifting step to further prevent mixing of intermittent oscillatory components ^136^. Here, we used an iterative method, “tailored mEMD” (tmEMD; https://github.com/cjcw/tmEMD), to obtain in an unsupervised manner the set of masking frequencies used for each brain region in each animal, using the tetrode channel whose (z-scored LFP) power spectral density had the smallest Euclidean distance to the median power spectral density computed over all tetrodes across animals for that region. In the first iteration, we randomly generated 200 sets of masking frequencies. We then applied mEMD on the LFPs with each set of masking frequencies and computed a mixing score, defined as the absolute maximum of all pairwise correlations between the resulting IMFs. For each subsequent iteration, the ranges for the next sets of masking frequencies were restricted to the ranges observed in the top 10 current mixing scores. This iterative process converged to a set of masking frequencies that yielded the lowest mixing score for each brain region. As well as measuring mode mixing, the tmEMD algorithm also computed the between-animal consistency scores. Following the completion of the algorithm, each set of mask frequencies was given a score, defined as the mean of its mode mixing and consistency ranks. This score was then used to define the region-tailored mask frequencies to be used for mEMD. To assess the spectral consistency of the LFP signals across individual tetrodes, the PSDs of each tetrode IMF were combined to form a feature vector for each tetrode. We then applied Principal Component Analysis on the corresponding [tetrode x feature elements] matrix. To the first four principal components of this matrix (>97.6% of the variance explained), we applied t-distributed stochastic neighbor embedding (tSNE) ^118^ to visualize the spectral similarity of tetrode oscillatory components across animals and regions (Figure S1A).

#### Cross-network activity barcode detection, tracking, and cocaine-paired memory score

To identify motifs of coordinated oscillatory activities within and between brain regions, we extracted network patterns of high-order (i.e., beyond one pair) IMF co-engagement (https://github.com/cjcw/barcode). To focus on short-lived, transient processes, we considered the IMFs with main frequencies between 12 and 125 Hz (Figure S2B). This selection yielded four signals for PFC, NAc, Amy and Hpc (i.e., 4 IMFs per region) and three IMF signals for VTA (Figure S2B; n=19 total IMFs). We computed the time course of each IMF amplitude (Figure S2C, *upper panel)* using the Hilbert transform (scipy.signal.hilbert) and normalized each instantaneous amplitude by its standard deviation. We thus obtained a time-series matrix where the amplitude of each IMF had unitary variance (Figure S2C, *lower panel*). At each given time *t*, we defined vectors ***a**_t_* that contained the normalized instantaneous amplitudes of these IMFs spanning the five brain regions (Figure S2D). We represented the set of instantaneous IMF amplitude co-modulation at time *t* by taking the outer product of its vectors ***a**_t_* only considering entries above and off the main diagonal such that all IMF pairs subsequently used were unique (i.e., each cross-IMF pair was only used once) and bipartite (i.e., no pairs were formed by a single IMF with itself) (Figure S2D,E). We used these entries obtained from the outer product of vectors ***a**_t_* as the elements of vector ***x***_t_, which therefore contained the set of instantaneous IMF co-engagement values (n=171 cross-IMF pairs) at time *t* (Figure S2D,F).

To next detect cross-network motifs of coordinated oscillatory activities that consistently occur over time (the cross-network “barcodes” of co-engaged oscillatory activities), we applied Independent Component Analysis on a feature matrix consisting of ***x**_t_* vectors sampled every 250 ms (Figure S2D,F). Each extracted independent component is a weight vector with individual elements corresponding to the amplitude co-modulation for a given pair of IMFs (Figure S2F). The strength of a given barcode at time *t* was then defined as the dot-product between the weight vector of its independent component and the instantaneous IMF co-engagement vector ***x**_t_*. This computation was performed on the co-engagement time-series matrix (derived from the non-normalized amplitude matrix) at original time resolution (1.25 kHz) to track the instantaneous barcode strength (Figure S2G). For visualization purposes, each barcode weight vector was converted to a square matrix to depict its corresponding network motif of IMF co-engagement (Figure S2H,I). The barcodes obtained in this study were detected across all animals, and cross-validated to assess between-animal consistency (Figure S2J).

To identify the barcodes whose strength modulation showed the strongest signatures of cocaine-paired memory retrieval (i.e., increased expression strength in recall and renewal compared to pre-test and extinction), we computed a barcode cocaine-paired memory retrieval (CPMR) score (Figures 2B and S2K). For each barcode, we calculated the time course of its z-scored expression strength in each test stage) using 1-s time bins while the animal explored the test enclosure (e.g., Figure 2A, bottom panel and Figure S2G). We then calculated the cocaine-minus-saline LED zone effect size ^137^ between the obtained barcode strength distributions for each of the four task stages in the test enclosure (i.e., pre-test, recall, extinction and renewal) during active locomotion (speed>2 cm.s^-1^) bins. This procedure yielded a stage-wise vector containing the four barcode expression strength effect sizes for the difference between cocaine- versus saline-paired LED activation zones. The corresponding barcode CPMR score was obtained as the dot-product between this 4-element barcode strength vector and the 4-stage binary CPMR vector representing the task stages in the test enclosure (where element 0 corresponds to no cocaine-paired memory retrieval in the pre-test and extinction stages and element 1 corresponds to cocaine-paired memory retrieval in the recall and renewal stages). To measure the contribution of a particular frequency band to a given barcode (Figure S3E), a binary barcode vector mask was defined, where any pairwise interaction element involving that frequency band corresponded to a value of 1; and all other elements, 0. The contribution was then taken as the dot product between this mask vector and the barcode vector, divided by the sum of the mask vector.

#### Beta bouts and amplitude modulation strength

To detect bouts of high-amplitude beta in each recorded brain region, we used the extracted beta-band IMFs to isolate individual beta cycles. Each single cycle was defined by its ascending zero-crossing, positive-peak and descending zero-crossing; being in between two beta troughs, and with an additional ascending zero-crossing being detected after the second trough, before the next peak. The mean amplitude of each detected beta cycle was calculated from its instantaneous amplitude time course. We defined a beta bout as a chain of at least 5 consecutive cycles that all have a mean amplitude value above the 50th percentile of cycle amplitude values. The start-trough of the first cycle in the chain defined the beta bout start, and the end-trough of the last cycle in the chain defined the beta bout end.

To compare the modulation strength of the amplitude of a given time series by the phase of 4-Hz oscillations (Figure 4), we calculated the average z-scored amplitude within 32 equally spaced phase bins. The modulation strength was then defined as the amplitude difference between the most- and least-preferred bin indices. These phase modulation scores were used for statistical analysis relating to Figure 4B and to compare the modulation of the beta-band and (slow, mid, fast) gamma-band signals in Figure S5D-G.

To assess the effect of closed-loop VTA optogenetic silencing on cross-regional beta amplitudes (Figure 7F,J), each animal-region beta amplitude signal was z-scored and the average response with respect to the phase-triggered pulse onset was computed and compared between the recall and the renewal stages. Note that there was no actual light delivery during the recall stage (i.e., laser not powered), but an equivalent TTL pulse timestamp used for this comparison. The average amplitude was taken from 0 to 125 ms (TTL pulse-on) and from -125 to 0 ms (TTL pulse-off) windows. The difference between pulse-on minus pulse-off amplitudes is reported.

#### Neuronal spiking activity

To assess spiking correlates of beta-band signals, the instantaneous firing activity of recorded neurons was triggered by the troughs of the beta bouts detected in either PFC, NAc, Amy, or CA1 Hpc (using reference troughs separated by at least 250 ms; Figure 3B). To initially gather further evidence for cross-regional beta-band modulation of firing activity, we calculated for each neuron its spike-phase coherence (mean phase vector length) to detected beta signals during beta-bouts as: *abs* (*mean* (*e^p.j^*)), where *p* is the vector of spike-sampled phases (in radians) and $j=\sqrt{-1}$. This coherence value was compared to a null distribution of coherence values, obtained by shuffling the beta bout windows, preserving both bout durations and inter-bout intervals. With this approach, a cell was deemed to be strongly beta-modulated if its beta bout coherence value was above the 95^th^ percentile of the null distribution (PFC n=23/421, NAc n=25/292, Amy n=3/247, Hpc n=13/477, and VTA n=19/132 cells). We then used the Rayleigh test (astropy 2.0.2) to assess modulation significance. The proportions of cells that are significantly beta-modulated across regions is reported in Figure S4A.

To explore the heterogeneity of the VTA cell population, we computed the average spike waveform for each recorded neuron using the tetrode channel with the highest spike waveform amplitude. This average waveform was then normalized by its maximum absolute value to construct a feature matrix (cells x waveforms). This matrix contained the spike waveforms of VTA cells recorded in wild-type mice during non-optogenetic experiments (i.e., where the molecular identity of recorded neurons was not optogenetically tested), along with the spike waveforms of both dopaminergic and glutamatergic VTA neurons recorded during light-off periods in DAT-Cre and Vglut2-Cre mice (where we further combined neuronal recordings with optogenetic stimulation for optogenetic identification; see also Figure S7A-H). We applied Principal Component Analysis on this feature matrix and used the first seven principal components (explaining > 95% of the variance) as feature elements to Uniform Manifold Approximation and Projection (UMAP) ^138^ for subsequent non-linear, dimensionality reduction. The projections of each VTA cell onto this UMAP embedding are shown in Figure 5A, and we obtained the VTA cell clusters using k-means (scikit-learn 0.24.4).

To assess whether the firing activity of a given cell was optogenetically modulated by light delivery (Figures 5, 6 and S7), the laser pulse times were randomly split into two halves (half-1 and half-2). For each half-set of laser pulse times, we computed the spiking response of each VTA cell with respect to the onset of light delivery, averaging over all laser pulses and using 2-ms time bins. For a given cell, the time bin corresponding to the peak firing response in half-1 was used to sample the binned firing response in half-2. For a VTA cell to be determined as opto-tagged, this firing response had to be above the 99th percentile of binned firing response values observed between -1000 to -500 ms for both laser pulse sets, and the time corresponding to the maximum response evoked by half-2 had to be within 6 ms of the laser onset. For a cell to be considered as entrained by optogenetic activation of VTA Vglut2 cells, its maximum half-2 firing response (sampled by the peak response for half-1) had to be above the 95^th^ percentile of values –1000 to –50 ms. Number of significantly entrained cells: PFC: 40/260; NAc: 57/177; Amy: 25/217; Hpc: 48/309; VTA 41/70 (9 of which were opto-tagged).

To compare the firing coupling of individual neuron members of a given VTA cell cluster (Figure 5A) to VTA 4-Hz oscillations, we computed population coherence percentile plots (Figure 5B). For each VTA cluster, this analysis allows visualizing the relative representation of spike-phase coherence values (see above) to the phase of VTA 4-Hz oscillations (y-axis) above a given percentile threshold (x-axis). To do so, we first calculated the whole-population percentile values corresponding to each single-neuron spike coherence observation in that cluster, then calculating the proportion of spike coherence values above a given whole-population percentile threshold. To test the significance of the over- or under-representation along the obtained curve, we computed a series of 1,000 “null curves” by repeatedly shuffling the group labels of all cells. For each VTA cell cluster, the corresponding population coherence percentile curve was deemed to represent significant over- or under-representation if falling outside of the 99% confidence limits of the null distribution. For each VTA cell cluster, the colored thicker line in its population coherence percentile curve represents significant over- or under-representation (Figure 5B).

To assess cross-regional spiking entrainment by VTA glutamatergic cell activation, we transduced VTA Vglut2-expressing neurons with the Cre-dependent viral construct encoding the blue light (473 nm)-driven neural activator channelrhodopsin-2 (ChR2) in mice expressing the Cre-recombinase under the control of the vesicular-glutamate transporter-2 (Vglut2) promoter (Figure 6). Optogenetic stimulation of these VTA^Vglut2^::ChR2 neurons was then obtained using 473-nm light 20-ms pulse delivery to VTA at random intervals (between 1 and 10 seconds) while performing ensemble recordings in PFC, NAc, Amy, Hpc and VTA. Neuronal spiking responses to light delivery were assessed as described above. The spiking responses of these cells (triggered by VTA light onset) were z-scored and used to construct the histogram and raster plots in Figure 6C.

#### LFP spectrograms

For triggered spectrogram analyses (Figures 4D, 5D, 6D and S6B,F), the LFP of a given region was first z-scored and the spectrogram then computed using a set of complex Morlet wavelets (using scipy.signal.morlet function from the scipy 1.3.1 python module) with main frequencies from 2–200 Hz, with 1-Hz steps up to 100 Hz and 5-Hz steps up to 200 Hz. To assess the changes in beta power relative to VTA 4-Hz troughs between LED activation zones across task stages, for each animal and test-stage, the resulting triggered spectral responses for saline- and cocaine-LED activation zones were z-scored for each frequency (taking the mean and S.D. over both zone spectrograms) before averaging over animals. Changes in beta power were then quantified by taking the median time of maximal beta across animal-zone responses (for each stage) to reference the z-scored beta power for each frequency within the beta range (Figures 4D,E and S6B,F). To explore changes in cross-regional spectral power following spiking incidences of a given VTA cell cluster (Figure 5D), we used the spike train of each cell member of that cluster, considering both spikes discharged in isolation and the first spike discharged in a burst. A minimum of 20 spikes were required for this spike train to be used for triggering the average spectrogram responses centered around each spike for each of the recorded regions. As a control, the instantaneous speed of the animal was binned into 100 percentile bins. Each of the selected spikes were then randomly assigned a new speed bin-matched time, ensuring this did not correspond to a real spiking event for that cell. For each cell type, the corresponding triggered spectrograms of the real and speed-controlled spikes were averaged to compute the mean spectral response, and then z-scored for each frequency wavelet to assess changes with reference to spike times. For each cell, the speed-controlled spectrogram response was then subtracted from the original to produce the spike-triggered spectrogram responses, which were averaged over cells in that cluster. For the light pulse-triggered spectrograms (Figure 6D), the average of each spectral response, centered by the onset of each pulse, was taken and averaged over animals. All spectrograms were plotted using the matplotlib.pyplot.contourf function.

#### Anatomy

Mice were deeply anesthetized with isoflurane/pentobarbital and transcardially perfused with PBS followed by cold 4 % PFA dissolved in PBS. The brains were extracted, kept in 4% PFA for 24 h, and sliced into 50 μm thick coronal sections. Free-floating sections were rinsed extensively in PBS with 0.25 % Triton X-100 (PBS-T) and blocked for 1 h at room temperature in PBS-T with 10 % normal donkey serum (NDS). Sections were then incubated at 4°C for 48 h with primary antibodies (chicken anti-GFP, 1:1000, Aves Labs, cat# GFP-1020, RRID: AB_10000240; guinea pig anti-TH, 1:2000, Synaptic Systems, cat# 213-104, RRID: AB_2619897; rabbit anti-Vglut2, 1:1000, Synaptic Systems, cat# 135-403, RRID: AB_887883) diluted in PBS-T with 3 % NDS (“blocking solution”). After that, sections were rinsed three times for 10 min in PBS and incubated for 24 h at 4°C in secondary antibodies (donkey anti-chicken Alexa Fluor 488, 1:500,Jackson ImmunoResearch, cat# 703-545-155, RRID: AB_2340375; donkey anti-guinea pig Cy3, 1:1000, Jackson ImmunoResearch, cat# 706-165-148, RRID: AB_2340460; donkey anti-rabbit Alexa Fluor 647, 1:500, Jackson ImmunoResearch, cat# 711-605-152, RRID: AB_2492288) diluted in PBS-T with 1 % NDS. This step was followed by three rinses for 15 min in PBS. Sections were then incubated for 1 min with 4’,6-diamidino-2-phenylindole (DAPI; 0.5 μg/ml, Sigma-Aldrich, cat# D8417) diluted in PBS to label cell nuclei, before undergoing three additional rinse steps of 10 min each in PBS. Sections were finally mounted on slides, cover-slipped with Vectashield mounting medium (Vector Laboratories) and stored at 4 °C. Images were acquired on a laser scanning confocal microscope (LSM 880/Axio Imager, Zeiss).

#### Data and Statistical analyses

Data and statistical analyses were performed in Python 3.6 (https://www.python.org/downloads/release/python-363/), using the packages scipy ^139^, numpy ^140^, matplotlib ^141^, seaborn ^142^, pandas ^143^, scikit-learn ^144^. Bar plots report group mean ± s.e.m., unless stated otherwise. All statistical tests related to a symmetric distribution were performed using two-sided using Gardner-Altman plots (to compare 2 groups) and Cumming plots (for more groups) as described in the Data Analysis with Bootstrap-coupled ESTimation (DABEST) statistics framework ^145^. These Difference Estimation plots allow visualizing the effect size by plotting the data as the mean or median difference between one of the groups (the left-most group of each plot, used as group-reference) and the other groups (to the right, along the x-axis of each plot). To estimate the effect size of unpaired observations from distributions *A* and *B*, the empirical mean difference is first calculated: Δ = A-B. For unpaired analyses (Figure 2, S3D,E, S6B,F), the group mean difference Δ is then subtracted from a series of null mean difference estimates, obtained by shuffling the group labels and then bootstrapping (n=5,000; unless stated otherwise) from each of the shuffled distributions. This shuffling operation is repeated 10,000 times to obtain a probability distribution estimate of the true mean difference. Paired mean difference estimation plots (Figures 1, 3, 4, 6, 7, S1, S3C, S4, S5 and S6A,E) are computed in a similar fashion whereby for each shuffle, paired labels are randomly swapped or preserved with equal chance. For each estimation plot: (*i*) the upper panel shows the distribution of raw data points for the entire dataset, superimposed on bar-plots reporting group mean±SEM, unless stated otherwise; and (*ii*) the lower panel displays the difference between a given group and the (left-most) group-reference, computed from 5,000 bootstrapped resamples and with difference-axis origin aligned to the mean or the median of the group-reference distribution. For each estimation plot: *black-dot*, mean (for normal distributions) or median (for skewed distributions) as indicated; *filled-curve*: bootstrapped sampling-error distribution; *black-lines*, error bars representing 95% confidence intervals (i.e., the 2.5th and 97.5th percentiles). For two-sided statistical tests, the percentile thresholds used are 2.5; 0.05; and 0.0005 for p < 0.05; p < 0.01; and p < 0.001, respectively. If a Bonferroni correction was used, then these percentiles were divided by the number of comparisons.

### Key Resources Table

**Table:** 

REAGENT or RESOURCE	SOURCE	IDENTIFIER
**Bacterial and Virus Strains**		
rAAV2-EF1a-DIO-hChR2(H134R)-eYFP-WPRE UNC	UNC Vector Core	n/a
rAAV2-CAG-FLEX-ArchT-GFP	UNC Vector Core	n/a
rAAV2-CAG-FLEX-GFP	UNC Vector Core	n/a
AAVrg-EF 1a-DIO-FLPo-WPRE-hGHpA	Zingg B. et al ^124^	Addgene, Catalog #87306-AAVrg
AAV5-EF1a-fDIO-eYFP-WPRE	UNC Vector Core	n/a
pAAV-hSyn-Con/Fon-ArchT-eYFP	Pavel Perestenko, MRC BNDU	PPV331B
pAAV-CamKII-ArchT-GFP	Han X. et al ^125^	Addgene, Catalog #37807
pAAV-hSyn-Coff/Fon-hChR2(H 134R)-eYFP	Fenno L.E. et al ^126^	Addgene, Catalog #55648
pAAV-hSyn-Coff/Fon-ArchT-eYFP	Pavel Perestenko, MRC BNDU	PPV321B
**Experimental Models: Organisms/Strains**		
Vglut2-ires-cre B6J.129S6(FVB)- Slc17a6tm2(cre)Lowl/MwarJ	Jackson Laboratory	IMSR_JAX:028863
DAT-ires-cre B6.SJL-Slc6a3tm1.1(cre)Bkmn/J	Jackson Laboratory	IMSR_JAX:006660
DAT-P2A-Flpo Slc6a3em1(flpo)Hbat/J	Jackson Laboratory	IMSR_JAX:035436
C57BL/6J mice	Charles River	632
**Software and Algorithms**		
Intan RHD2000	Intan Technologies	RHD2164
POLYtrack	Imetronic	n/a
Positrack	Kevin Allen	n/a
OscillTrack	McNamara C.G. et al. ^130,131^	https://doi.org/10.5287/bodleian:qa9ngXrzr
Empirical Mode Decomposition in Python	Quinn A.J. et al. ^134^	n/a
Tailored Masked Empirical Mode Decomposition	Charlie Clarke-Williams, this study	https://doi.org/10.5281/zenodo.10351412
Cross-network LFP barcodes	Charlie Clarke-Williams, this study	https://doi.org/10.5281/zenodo.10351398
Kilosort via SpikeForest	Magland J.F. et al. ^132^; Pachitariu M.^133^ et al.	n/a
**Other**		
12um tungsten wires	California Fine Wire	M294520
Optic fibers	Doric lenses	MFC_200/230- 0.37_10mm_RM3_FLT
Head-stage amplifier	Intan Technologies	RHD2164
561nm diode-pumped solid-state laser	Laser 2000	CL561-100
473nm diode-pumped solid-state laser	Laser 2000	CL473-100

## Supplementary Material

Figure S1

Figure S2

Figure S3

Figure S4

Figure S5

Figure S6

Figure S7

Table S1

Supplementary figures legends

## Data Availability

The electrophysiology dataset reported in this study is not yet openly available, as it is being used in ongoing projects. We welcome enquiries for sharing this as part of a collaboration, please contact david.dupret@bndu.ox.ac.uk. All original code has been deposited at Zenodo and is publicly available as of the date of publication. DOIs are listed in the key resources table. Any additional information required to reanalyze the data reported in this work paper is available from the Lead Contact upon request.
