# Supplementary figures and images for: Coordinating brain-distributed network activities in memory resistant to extinction
Source: Cell. Author manuscript; Available in PMC 2024 Jan 24. (PMC7615560; doi:10.1016/j.cell.2023.12.018)

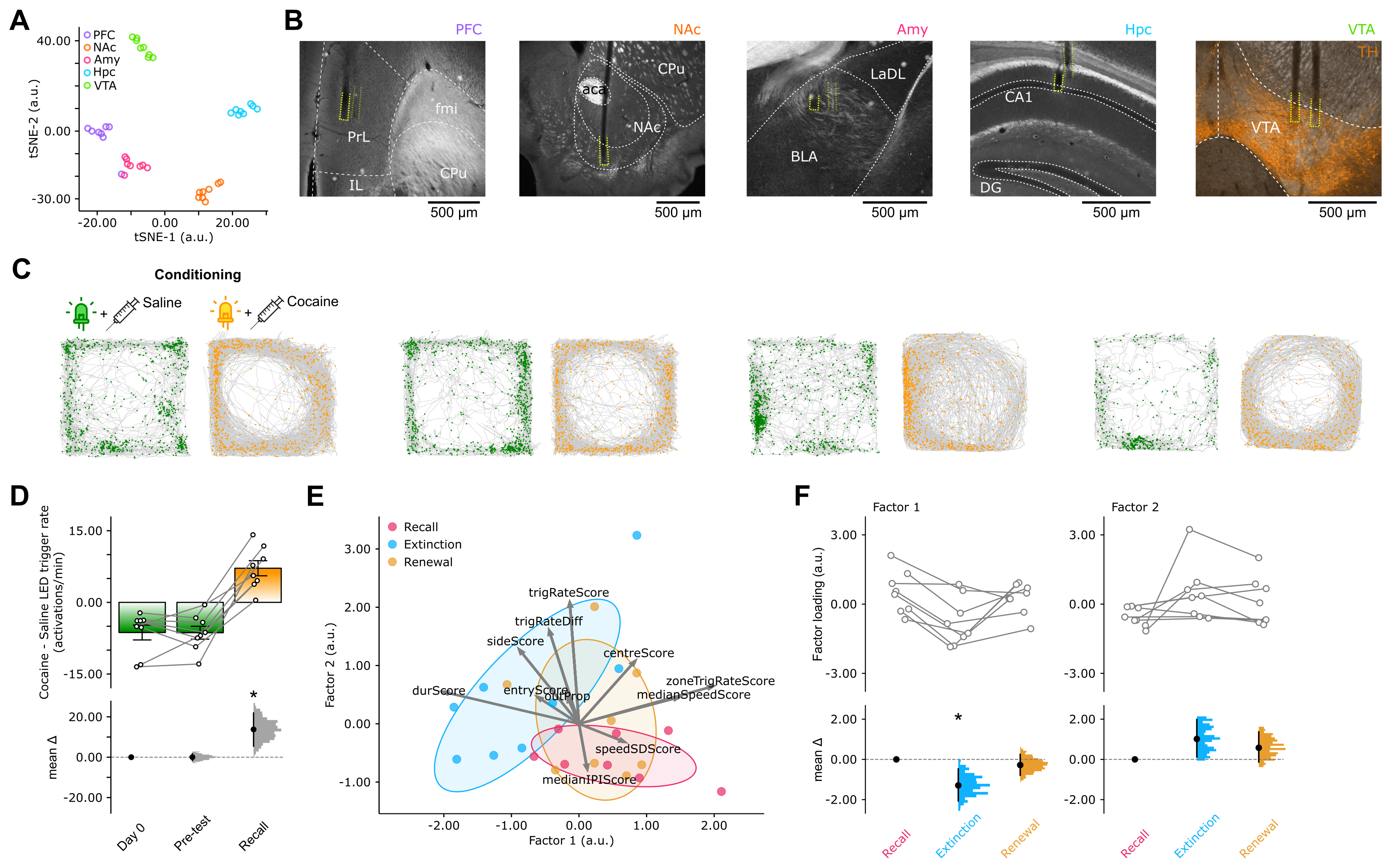

Supplement: Figure S1 [file EMS193001-supplement-Figure_S1.tif]

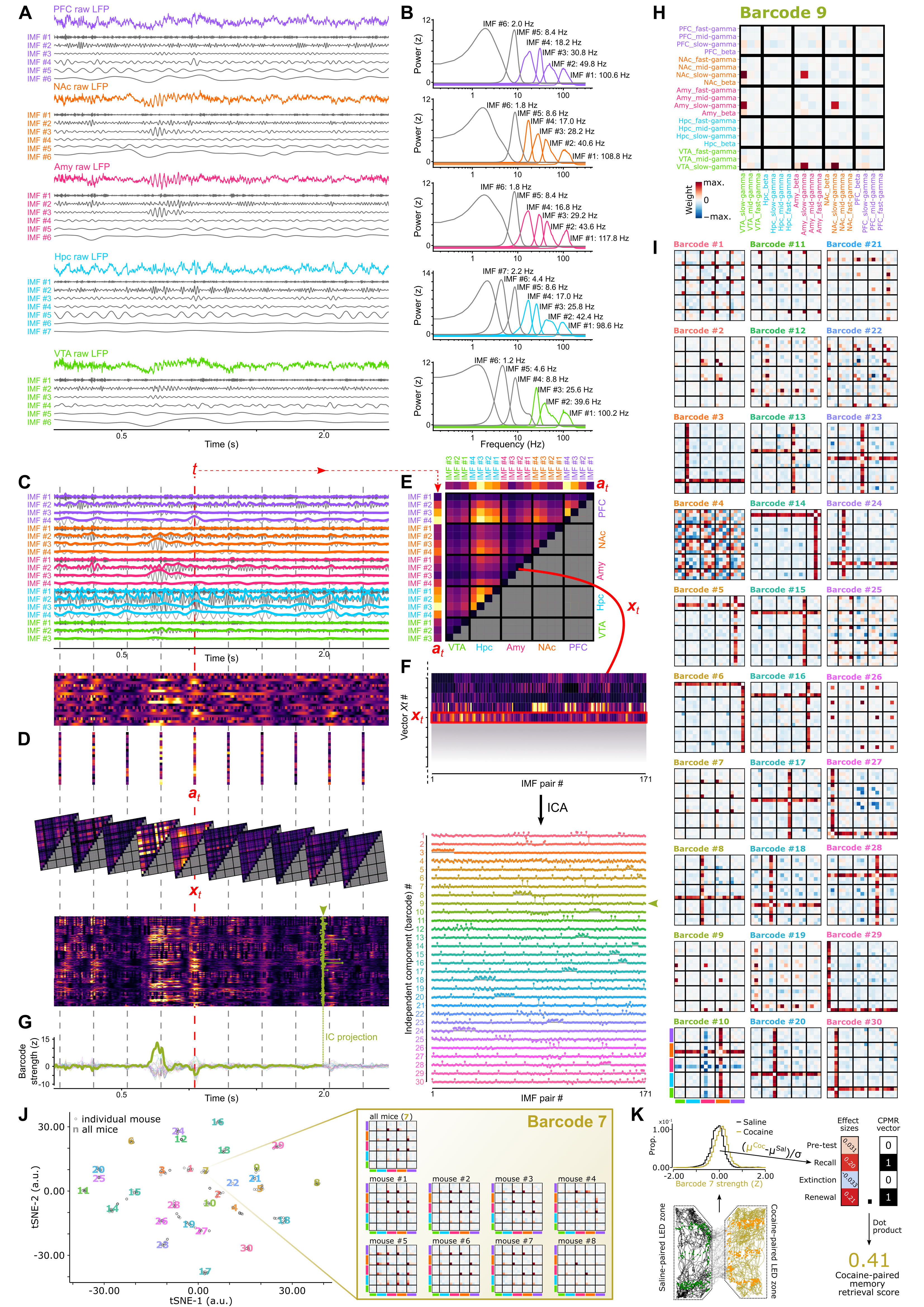

Supplement: Figure S2 [file EMS193001-supplement-Figure_S2.tif]

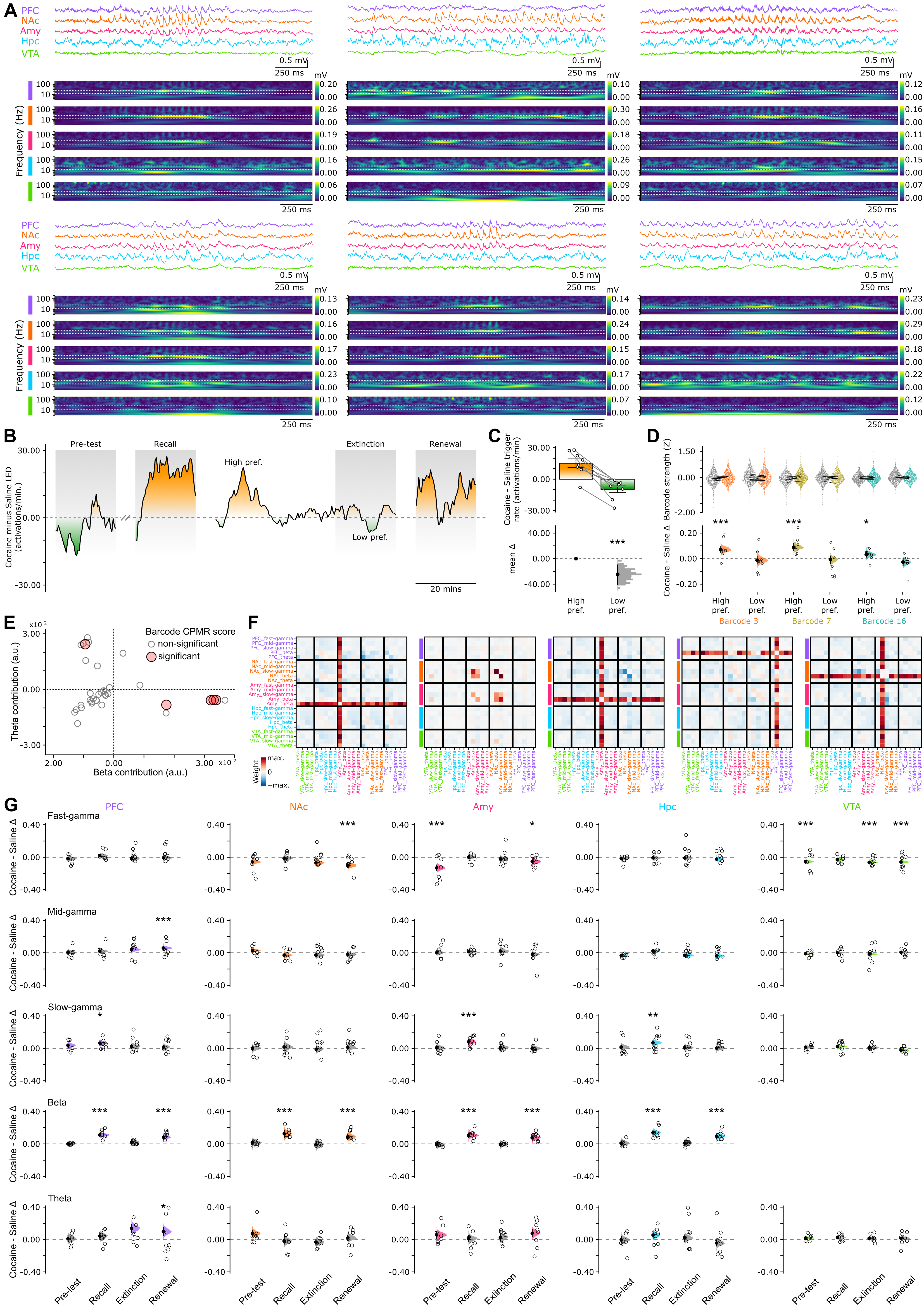

Supplement: Figure S3 [file EMS193001-supplement-Figure_S3.tif]

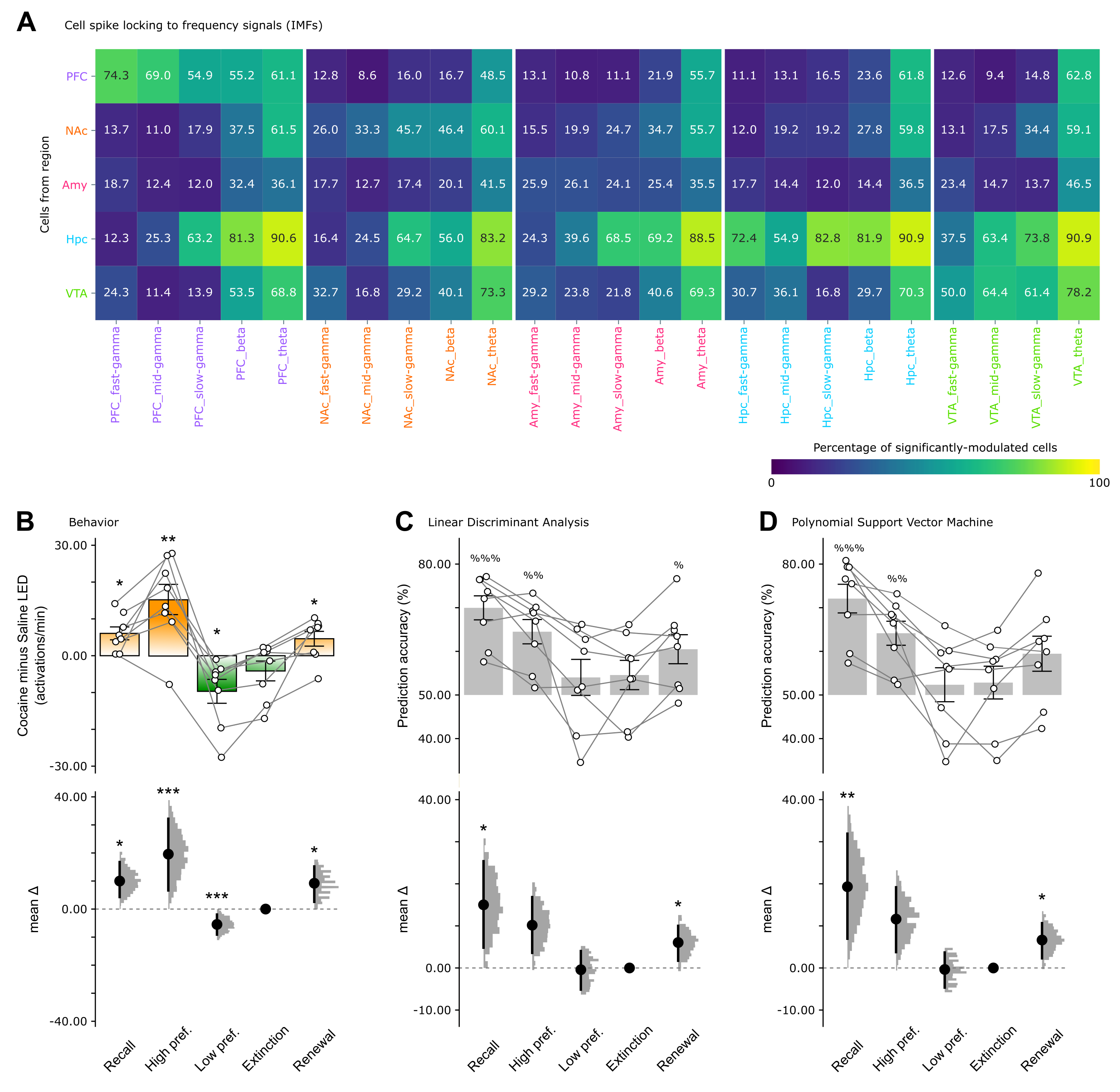

Supplement: Figure S4 [file EMS193001-supplement-Figure_S4.tif]

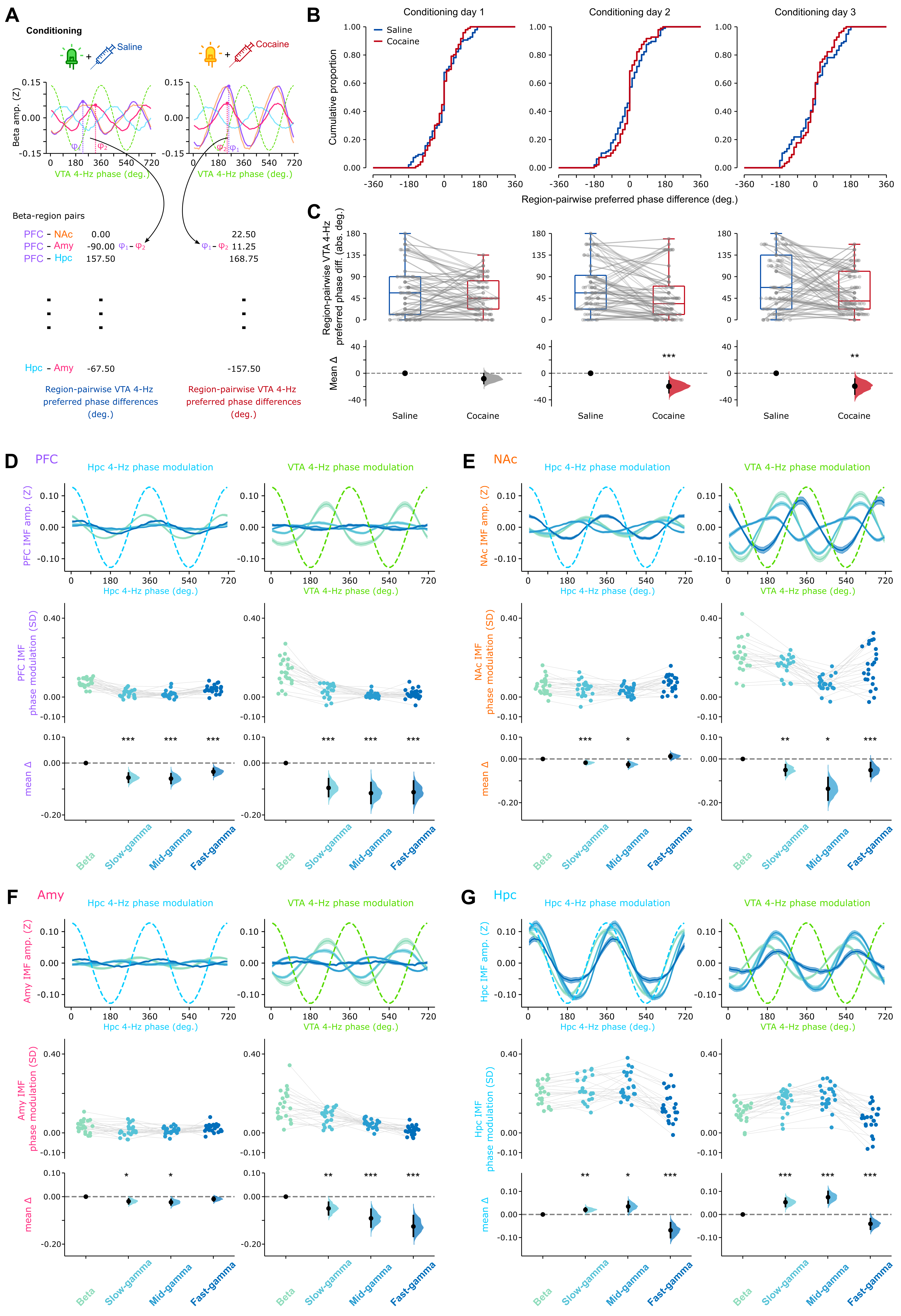

Supplement: Figure S5 [file EMS193001-supplement-Figure_S5.tif]

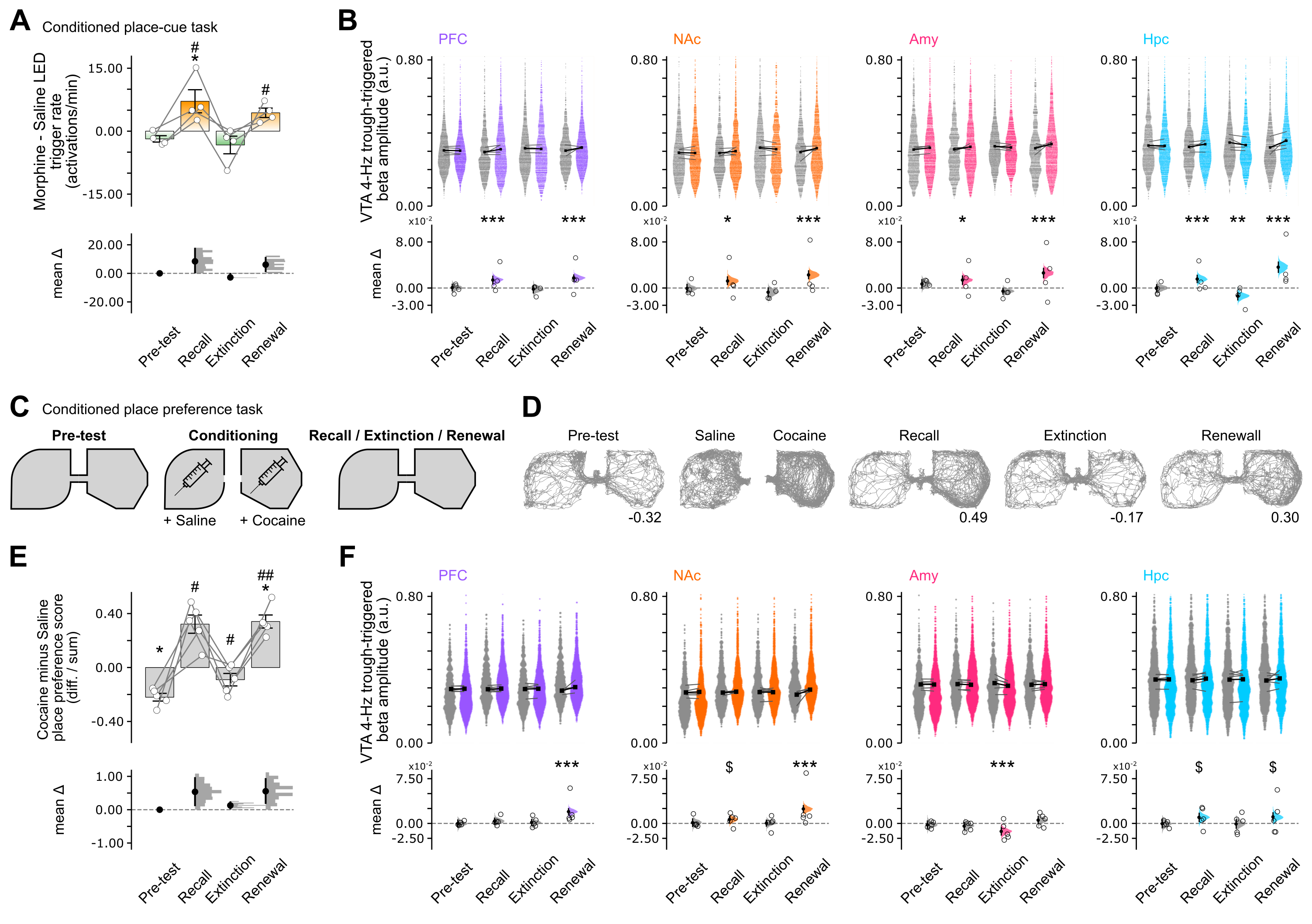

Supplement: Figure S6 [file EMS193001-supplement-Figure_S6.tif]

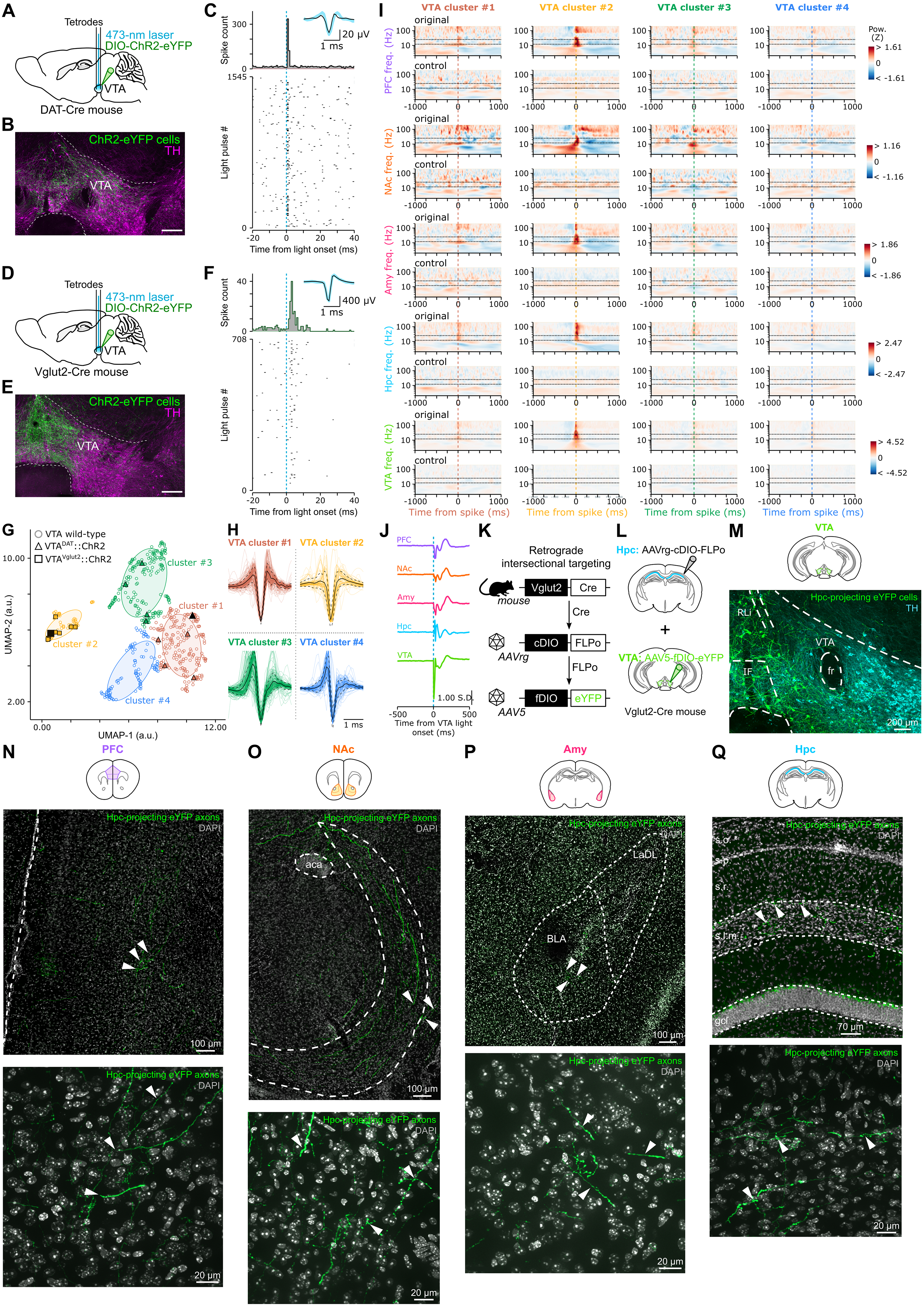

Supplement: Figure S7 [file EMS193001-supplement-Figure_S7.tif]
