# Supplementary material for: Coordinating brain-distributed network activities in memory resistant to extinction
Source: Cell. Author manuscript; Available in PMC 2024 Jan 24. (PMC7615560; doi:10.1016/j.cell.2023.12.018)
Supplement: Table S1 [file EMS193001-supplement-Table_S1.docx]

**Supplemental table**

**Table S1: Behavioral metrics for Factor Analysis of cocaine-biased behavior (related to STAR Methods and Figure S1E,F).**

| **Metric** | **Description** |
| --- | --- |
| trigRateScore | A preference score equals to the number of cocaine-paired LED trigger events minus that of saline-paired LED trigger events, divided by the sum: $trigRateScore= \frac{{trig}^{Coc}- {trig}^{Sal}}{{trig}^{Coc}+ {trig}^{Sal}}$ |
| trigRateDiff | The difference between the number of cocaine-paired LED activations minus that of saline-paired LED activations: $trigRateDiff= {trig}^{Coc}- {trig}^{Sal}$ |
| zoneTrigRateScore | A preference score for cocaine-paired LED activation rate (i.e., ${trig}^{Coc}$ divided by time spent in cocaine zone ${time}^{Coc}$) versus saline-paired LED activation rate: $zoneTrigRateScore= \frac{\frac{{trig}^{Coc}}{{time}^{Coc}}-\frac{{trig}^{Sal}}{{time}^{Sal}}}{\frac{{trig}^{Coc}}{{time}^{Coc}}+\frac{{trig}^{Sal}}{{time}^{Sal}}}$ |
| durScore | A preference score for the time spent in cocaine zone versus that in saline zone: $durScore= \frac{{time}^{Coc}-{time}^{Sal}}{{time}^{Coc}+{time}^{Sal}}$ |
| outProp | The proportion of time spent in the central area (i.e., not in the saline- or cocaine- zones) |
| medianSpeedScore | A preference score for the median speed in the cocaine zone versus that in the saline zone: $medianSpeedScore= \frac{{speed}^{Coc}- {speed}^{Sal}}{{speed}^{Coc}+ {speed}^{Sal}}$ |
| speedSDScore | A preference score using the speed standard deviation in the cocaine zone versus that in the saline zone: $speedSDScore= \frac{{SD}^{Coc}- {SD}^{Sal}}{{SD}^{Coc}+ {SD}^{Sal}}$ |
| entryScore | A preference score for the number of entries in the cocaine versus saline zone: $entryScore= \frac{{entry}^{Coc}- {entry}^{Sal}}{{entry}^{Coc}+ {entry}^{Sal}}$ |
| sideScore | A preference score for the number of cocaine-paired LED trigger events on either of the side quadrant boundaries versus saline-paired LED trigger events: $sideScore= \frac{{trigSide}^{Coc}- {trigSide}^{Sal}}{{trigSide}^{Coc}+ {trigSide}^{Sal}}$ |
| centreScore | A preference score for the number of cocaine-paired LED trigger events on the centre quadrant boundary versus saline-paired LED: $centreScore= \frac{{trigCentre}^{Coc}- {trigCentre}^{Sal}}{{trigCentre}^{Coc}+ {trigCentre}^{Sal}}$ |
| medianIPIScore | A preference score for the median of inter-pulse intervals (i.e., median time between LED trigger events) for the cocaine- versus saline-paired LED: $medianIPIScore= \frac{{IPI}^{Coc}- {IPI}^{Sal}}{{IPI}^{Coc}+ {IPI}^{Sal}}$ |
