# Supplementary material for: Coordinating brain-distributed network activities in memory resistant to extinction
Source: Cell. Author manuscript; Available in PMC 2024 Jan 24. (PMC7615560; doi:10.1016/j.cell.2023.12.018)
Supplement: Supplementary figures legends [file EMS193001-supplement-Supplementary_figures_legends.docx]

# Supplementary figures legends

**Figure S1. Consistency of tetrode LFP signals and behavior in the conditioned cue-place preference task (related to Figure 1).**

**(A,B)** Assessment of tetrode position. For the recordings, we used the spectral content of the LFPs from each tetrode as an electrophysiological readout of their position, adjusting it to the target region by using the tetrode cannula-holding screw that enabled their independent movement along the vertical axis. The spectral content of the LFP signals was consistent for tetrodes targeting the same region across animals (**A**; data visualised using tSNE, with each color-coded data point representing the tetrode targeting a given region in one animal; see STAR methods). After the recording experiment, tetrode positions were confirmed by histology of anatomical tracks (**B**, coronal sections imaged using dark field transmitted light, with additional staining for tyrosine hydrolase on the VTA section; yellow thick dashed lines show tetrode tips; yellow thin dashed lines show neighbouring tracks with tips ending in other sections).

**(C)** Additional example animal paths(grey) during saline (left) versus cocaine (right) sessions of the conditioning stage. The green and orange dots overlaid on each path represent the animal’s position when the saline- and the cocaine-paired LEDs were automatically replayed, respectively.

**(D)** Stability of the initial place-LED preference across the first two days of the task. The difference in cocaine- minus saline-paired LED activations per minute is shown for the first 2-day stage (day 0 and pre-test) along with that in the recall stage. Negative numbers report preferred saline-paired LED activation; positive numbers report preferred cocaine-paired LED activation. Note that mice maintained across the first two days prior to conditioning their initial preference for one of the two place-LED sets. They then reverted this initial preference for the cocaine-paired LED during the recall stage following conditioning. The dataset is represented using a difference estimation plot to visualize the effect size for this cocaine-biased behavior. Upper panel: raw data points, with each set of three connected points reporting the difference between cocaine- minus saline-paired LED activation for one mouse across the first task session (day 0; before pre-test), pre-test, and recall; bar charts: average (mean±SEM) LED activation preference across animals. Lower panel: corresponding effect size, using the mean difference estimation for each day compared to day 0. *Black-dot*, mean difference; *filled-curve*: distribution of mean differences; *black-lines*, 95% confidence interval; ** p<0.01 paired permutation test compared to day 0, with Bonferroni correction for multiple comparisons.

**(E,F)** Factor analysis of mouse behavior during recall, extinction, and renewal.

To evaluate the existence of a common process underlying the multivariate nature of cocaine-biased behavior in the recall, extinction, and renewal stages of our task, we applied Factor Analysis for dimensionality reduction of a set of behavioral metrics related to animal’s response with respect to triggering cocaine- versus saline-paired LEDs in the test enclosure (see **Table S1**). (**E)** shows the factor analysis score and loading biplot. Each data point represents the score of the second factor versus the score of the first factor for one animal, color-coded according to recall, extinction, and renewal stages. Each arrow represents the loading of one behavioral metric on the factors. **(F)** shows the estimation plots to visualize the effect size in changes in animal factor loadings across recall, extinction, and renewal. Each set of three connected points in the upper panels show the raw loadings for each animal across the three task stages. The bottom panels show the paired mean difference estimation (effect size) compared to recall. *Black-dot*: mean difference estimate; *filled-curve*: distribution of mean difference estimates; *black-lines*: 95% confidence interval.

**Figure S2. Cross-brain-network activity barcodes: detection and consistency (related to Figure 2).**

**(A)** Example raw LFP traces (colored) with decomposed oscillatory components (IMFs) extracted using tailored masked Empirical Mode Decomposition shown underneath (grey) for each region.

**(B)** Power Spectral Density for each extracted IMF. IMFs with a main frequency within the 12-150Hz range (colored) were selected for barcode detection.

**(C)** Instantaneous amplitude time courses for each of the selected IMFs from each region (top). These amplitudes were normalized by their standard deviation to produce the amplitude time-series matrix (bottom).

**(D-F)** Amplitude vectors$\boldsymbol{a}_{t}$were sampled every 250ms **(D)** and converted to a co-engagement matrix by means of their outer product **(E)**. Elements within the off-diagonal of this matrix were used to define the vector $\boldsymbol{x}_{t}$, which represented all unique pairwise co-engagements of IMFs. These $\boldsymbol{x}_{t}$ vectors were used to construct a feature matrix (**F**, top), to which Independent Component Analysis (ICA) was applied, resulting in 30 independent components (“barcodes”) (**F**, bottom).

**(G)** The instantaneous strength of a given barcode vector (e.g., barcode #9) was calculated by projecting its weight onto the non-normalized co-engagement time-series.

**(H)** Each independent component vector was converted into a square matrix to visualize the co-engagement pattern it represented (e.g., barcode #9).

**(I)** Visualization of all barcodes detected across all animals (as example in **H**), which were used for the analyses in **Figure 2**.

**(J)** Consistency of barcode detection between animals. Barcodes were detected for each animal individually to examine the consistency of barcode detection between animals. This is visualized using the t-distributed stochastic neighbor embedding (tSNE) algorithm ^118^. *Open circles*, barcodes detected for individuals; *Colored numbers*, barcodes detected across the group data, as in **(H,I)**.

**(K)** Schematic depiction of cocaine-paired memory retrieval (CPMR) score formula. For a given animal, the barcode strengths are sampled over 1-s windows of active (speed>2 cm/s) exploratory behavior when the animal is in the saline- versus the cocaine-paired LED trigger zone in each test stage (pre-test, recall, extinction, and renewal). The effect size (difference of means, divided by standard deviation of all data) is then calculated, producing a test-stage-wise vector representing the four-stage barcode expression strength differences as cocaine-minus-saline effect sizes. To calculate the corresponding barcode CPMR score, the dot-product is taken of this 4-element barcode strength vector against the 4-stage binary vector representing the task stages in the test enclosure (i.e., the 4-element reference vector (0.0; 1.0; 0.0; 1.0) where element 0.0 corresponds to no cocaine-paired memory retrieval in the pre-test and extinction stages and element 1.0 corresponds to cocaine-paired memory retrieval in the recall and renewal stages).

**Figure S3. Beta-band signals, barcode strength during extinction and cross-network amplitude modulation through test stages (related to Figure 2).**

**(A)** Example bouts of beta-band rhythmic signals directly observable from the raw wide-band LFP traces of PFC, NAc, Amy and CA1 Hpc. For each example, shown are raw LFP traces (*top*; color-coded according to the region) and their corresponding spectrograms (*bottom*; with horizontal white dashed lines marking the beta-frequency range).

(**B-D**) Changes in beta-band barcode strength during the post-recall session progressing toward extinction. Shownisan example time course of cocaine-paired LED activation bias across the four stages in the test enclosure for one mouse (**B**) with test sessions labelled using gray fill along with the high- and low- preference epochs in the post-recall session progressing towards extinction; the corresponding assessment of the cocaine minus saline LED trigger preference (**C**), and the estimation plot (**D**) for changes in cocaine minus saline zone modulation strength for the three barcodes (# 3, 7 and 16) with the highest cocaine-paired memory retrieval scores (see **Figure 2**). (**D**) Top: beeswarm plots of barcode strength observations from 1-s windows when the mouse was active (speed > 2 cm s^-1^) in the saline- (gray dots) or cocaine- (colored dots) *gray lines*:mean zone strengths for each animal; Bottom: corresponding cocaine minus saline mean difference estimation distribution for each stage, obtained from the beeswarm data points above; *black-dot*: mean difference estimate; *filled-curve*: distribution of mean difference estimates; *black-lines*: 95% confidence interval; *open circles*: animal mean differences. *** p < 0.001; * p < 0.05: permutation test.

(**E,F**) Most of the LFP barcodes with significantly stronger expression in the cocaine-paired LED zone during recall and renewal feature high beta-band contributions, even when including theta band signals in their detection. (**E**) is a scatter plot showing the contribution of beta-band signals versus that of theta-band signals to each detected barcode (individual circles). Barcodes with significant cocaine-paired memory retrieval (CPMR) scores (akin to **Figure 2B**; see **Figure S2K** for details) are shown as larger, red-filled circles. Note that only 1 barcode with high theta contribution has a significant CPMR score while 4 barcodes with high beta contribution have significant CPMR scores. These significant barcodes are shown in (**F**).

**(G)** Estimation plots showing the cocaine-minus-saline LED zone difference in amplitude modulation for the oscillatory signals in the beta-band, (slow, mid, fast) gamma-band and theta-band oscillations (rows) of each region (columns) through test stages. Changes in the amplitude of each signal computed when mice enter in the cocaine- minus the saline-paired LED activation zones for each task test stage (pre-test, recall, extinction, and renewal) using 1-s time windows of active exploratory behavior. In line with the barcode analysis, the only significant amplitude modulation consistently observed across regions during both recall and renewal concerns beta oscillations (i.e., stronger beta amplitudes in cocaine-paired zones for all regions in both recall and renewal). While theta amplitudes can increase in cocaine-paired zones during recall or renewal for some regions, this enhancement can also occur during extinction. *** p < 0.001; ** p < 0.01; * p< 0.05: permutation test, with Bonferroni correction for multiple comparisons (n = 25).

**Figure S4. Neuronal spiking coupling to oscillatory signals and decoding of active place-LED set (related to Figure 3).**

**(A**) Spike locking to frequency signals detected across brain regions. To compare the extent to which cells from each region were locked to individual oscillations, we applied the Rayleigh test for non-uniformity to the spike-sampled phases of theta, beta, slow-gamma, mid-gamma and fast-gamma signals detected in each region. Numbers indicate the percentage of cells that are significantly modulated. For a cell to be included in this analysis, a minimum threshold of 200 spikes was imposed. To mitigate the effect of spike-leakage on coherence values ^119^, for local cell coupling to fast-gamma signals, the mean p-value across local reference tetrode not associated with that cell was used.

(**B-D**) The relationship between neuronal spiking and active place-LED set during the post-recall session progression towards extinction (**B**; see also Figure S3B) was assessed using linear discriminant analysis (**C**) and polynomial support vector machine (**D**) models. Each model was fitted to identify the active place-LED (set 1 versus set 2) from the ongoing population vectors of spike counts (using 100-ms windows) during recall and then applied in the high- and low- preference epochs of the post-recall session progressing towards extinction; the extinction session; and the renewal session. Top row: (**B**): *** p < 0.001; ** p < 0.01; * p < 0.05: repeated ANOVA for pairwise stage-stage (versus extinction) (**C,D**): %%% p < 0.01; %% p<0.01; % p < 0.05; $ p = 0.051: 1-sample t-test versus chance (50%). Bottom row (**B-D**): *** p < 0.01; ** p< 0.01; * p < 0.05: paired permutation test versus the extinction stage, with Bonferroni correction for multiple comparisons.

**Figure S5. Enhanced coordination of beta phase tuning to VTA 4-Hz under cocaine and preferential modulation of cross-regional beta signals by VTA 4-Hz (related to Figure 4).**

**(A-C)** The VTA 4-Hz coordination of beta activities detected in individual (PFC, NAc, Amy, and CA1 Hpc) regions improved across the 3-day cocaine conditioning. For each pair of brain regions, we computed the VTA 4-Hz preferred phase differences of corresponding regional beta amplitude signals. This was done for each animal, conditioning day, and conditioning session (saline versus cocaine). The VTA 4-Hz phase at which the beta amplitude was highest was used to sample the preferred phase, and then the differences between these preferred phases for each pairwise region combination were calculated to measure the strength of phase coordination by VTA 4-Hz for Saline or Cocaine (**A**). **B** shows the cumulative distributions of these preferred phase differences. Note the sigmoid is steeper for Cocaine observations in conditioning day 2/3, suggesting tighter phase-co-ordination under cocaine. **C** (top) shows the paired preferred phase difference observations for each animal-region-pair, with the box-and-whisker plot indicating the minimum and maximum values, interquartile-range and median with the paired mean difference estimation plot shown below. *Black-dot*: mean difference estimate; *filled-curve*: distribution of mean difference estimates; *black-lines*: 95% confidence interval. *** p < 0.001; ** p < 0.01: determined from the cocaine-minus-saline paired mean difference estimation of region pairwise VTA 4-Hz preferred phase differences.

**(D-G)** Phase modulation of beta, slow-, mid- and fast-gamma IMF amplitudes from PFC **(D)**, NAc **(E)**, Amy **(F)** and Hpc **(G)** by Hpc- (left) and VTA- (right) 4-Hz. Top of each panel: IMF amplitude as a function of 4-Hz phase; *thick line*, group mean; shaded error, ±SEM. Middle and bottom of each panel: paired Gardner-Altman estimation plot showing differences in phase modulation. *middle*, distribution of the raw data points, with each set of four connected points reporting the phase amplitude coupling for a given animal. *lower:* effect size, using the mean difference estimation for each signal amplitude compared to beta signal. *Black-dot*, mean difference estimate; *filled-curve*: distribution of mean difference estimates; *black-lines*, 95% confidence interval. *** p < 0.001; ** p < 0.01; * p < 0.05: determined from the paired mean difference estimation of phase modulation scores, versus beta, with Bonferroni correction for multiple comparisons.

**Figure S6. Changes in VTA 4-Hz related beta-band power in two additional paradigms (related to Figure 4).**

**(A-B)** Morphine conditioned cue-place preference paradigm. (**A**) Behavioral place-LED preference (same task layout as with cocaine, Figure 1C). Estimation plot showing the effect size for changes in morphine-biased behavior across test stages (as in Figure 1G). Upper panel: raw data points, with each set of four connected points reporting the difference between morphine- minus saline-paired LED activation for one mouse in each stage; bar charts: average (mean±SEM) LED activation preference. ## and ** p<0.01: repeated ANOVA for pairwise stage-stage (#: vs pre-test; *: vs extinction) interactions, with Bonferroni correction for multiple comparisons. Lower panel: corresponding effect size, using the mean difference estimation for each stage compared to pre-test. (**B**) Changes in VTA 4-Hz trough-triggered beta-band power between morphine-paired minus saline-paired LED activations over tests (as in Figure 4E). Top: each data point represents the beta amplitude triggered by individual VTA 4-Hz troughs for a given region (columns) for the saline- (gray) or morphine (colored) -paired LED zones, across stages. Datapoints were bootstrapped (n=10,000) for each animal-zone-stage distribution. *Gray lines*:means for each animal; *black squares/lines*: distribution average. Bottom: corresponding morphine minus saline mean difference estimation distribution. *Black-dot*: mean difference estimate; *filled-curve*: distribution of mean difference estimates; *black-lines*: 95% confidence interval. *** p < 0.001; ** p < 0.01: permutation test, with Bonferroni correction for multiple comparisons; n = 4 stages.

**(C-F)** Conditioned place preference (CPP) paradigm. (**C**) Task layout using five stages. **(D**) Example animal paths during each stage on a given day (numbers indicate CPP scores, measured as the difference in time spent in the cocaine- minus the saline-paired compartments, over the sum). (**E**) Estimation plot showing CPP score across test stages. Upper panel: raw data points, with each set of four connected points reporting the CPP score for one mouse in each stage; bar charts: average (mean±SEM) CPP score. ## p < 0.01; # and * p<0.05: repeated ANOVA for pairwise stage-stage (#: vs pre-test; *: vs extinction) interactions, with Bonferroni correction for multiple comparisons. Lower panel: corresponding effect size, using the mean difference estimation for each stage compared to Pre-test. (**F**) Changes in VTA 4-Hz trough-triggered beta-band power between cocaine- minus saline-paired compartments over test stages. Top: each data point represents the beta amplitude triggered by individual VTA 4-Hz troughs for a given region (columns) for the saline- (gray) or morphine (colored) -paired LED zones, across stages. Data points were bootstrapped (n=10,000) for each animal-zone-stage distribution. *Gray lines*:means for each animal; *black squares/lines*: distribution average. Bottom: corresponding cocaine minus saline mean difference estimation distribution. *Black-dot*: mean difference estimate; *filled-curve*: distribution of mean difference estimates; *black-lines*: 95% confidence interval. $ p < 0.05, before Bonferroni correction; *** p < 0.001, permutation test, before and with Bonferroni correction for multiple comparisons; n = 4 stages.

**Figure S7. Identification of VTA cell clusters and anatomical evidence for a divergent VTA Vglut2 neuronal pathway (related to Figures 5,6,7).**

**(A-F)** We recorded the spike waveforms of optogenetically identified VTA dopaminergic (**A-C**) and glutamatergic (**D-F**) cells by transducing them with the Cre-dependent viral construct encoding the blue light (473 nm)-driven neural activator channelrhodopsin-2 (ChR2) in mice expressing the Cre-recombinase under the control of the dopamine transporter (DAT) or the vesicular-glutamate transporter-2 (Vglut2), respectively; subsequently implanting VTA tetrodes combined with VTA optic fibers for blue light delivery. **B** and **E** show VTA coronal sections with ChR2-eYFP expression in VTA cell bodies (along with tyrosine hydroxylase staining) in a DAT^VTA^::ChR2 mouse (**B**) and a Vglut2^VTA^::ChR2 mouse (**E**). Scale bars = 200 microns. **C** and **F** show an example opto-tagged VTA cell recorded in a DAT^VTA^::ChR2 mouse (**C**) and a Vglut2^VTA^::ChR2 mouse (**F**). Each example shows the histogram of the total spike count with respect to VTA light onset (top), and the corresponding raster plot showing the spiking incidences over individual light pulse events (bottom). At the top-right of each histogram is the spike waveform of the optotagged cell during the laser pulse (*blue lines*) and when the laser was off (*black lines*).

**(G)** Electrophysiological clustering of VTA neurons. The shape of spike waveforms was used in a dimensionality reduction algorithm (UMAP) to identify clusters of VTA neurons. This approach was applied to VTA neurons recorded in quintuple-brain-region implanted wild-type mice (each ○ represents one wild-type VTA neuron), and to VTA neurons recorded and optogenetically identified (ChR2 opto-tagged) as dopaminergic (Δ) and glutamatergic (□) cells. The two opto-tagged cells colored in black (▲ and ■) correspond to the examples shown in **C** and **F**.

**(H)** Spike waveforms of each VTA cluster. For each VTA cluster: each colored line represents the average spike waveform shape of a given VTA cell member of that cluster; the thick black line represents the waveform average of that cluster; the dashed black lines represent the standard deviation. Spike waveforms normalized to maximum absolute amplitude. The spike waveform of optogenetically identified Vglut2 glutamatergic cells matched that of wild-type cells in cluster #2, which we thus designated as the VTA putative glutamatergic cell cluster.

The spike waveform of optogenetically identified DAT dopaminergic cells matched that of cells in cluster #1,3, which we thus designated as VTA putative dopaminergic cell clusters.

**(I)** For each individual region (PFC, NAc, Amy, Hpc and VTA) shown are the average spectrograms constructed from the raw LFPs (top row for each region display) with their corresponding speed-matched control spectrograms (bottom row of each region display), used to construct the spectrograms shown in **Figure 5D**. All spectrograms with respect to the discharge of action potentials by neurons from each VTA cluster (one cluster per column).

**(J)** Average LFP traces relative to optogenetic stimulation of VTA Vglut2::ChR2 neurons.

**(K,L)** Intersectional approach for viral vector-mediated retrograde tract tracing of VTA Vglut2 neuron projections. The neuronal expression of the eYFP fluorescent report protein is made conditional to the activity of two (Cre and FLPo) recombinases (**K**). First, an AAVrg construct containing the Cre-dependent FLPo transgene (cDIO-FLPo) is injected in one brain region (here, the Hpc) of Vglut2-cre mice for retrograde transduction of the Cre-expressing Vglut2 neurons projecting to this (AAVrg-injected) target region (**K,L**). The VTA of the same mice was then injected with the second, AAV5 construct (fDIO-eYFP) for FLPo-dependent eYFP expression (**L**).

**(M-Q)** Coronal sections from the VTA (**M**; along with tyrosine hydroxylase staining), PFC (**N**), NAc (**O**), Amy (**P**) and Hpc (**Q**) from the same Vglut2-Cre mouse injected as described in (**K,L**). Using this approach, we observed that targeting the dorsal Hpc allowed retrograde transduction of Vglut2 neurons located in the medial VTA (**M**; see also Figure 7A-C). These Hpc-projecting VTA Vglut2 neurons also send axonal projections to PFC (**N**), NAc (**O**) and Amy (**P**); axonal projections are also shown for Hpc (**Q**). This viral tract tracing thus shows that individual Vglut2 glutamatergic VTA neurons can project to multiple brain region targets, thereby constituting a divergent pathway that reaches all recorded brain regions from VTA. Cell nuclei stained with DAPI. Anterior cerebral artery (aca), Basolateral/Dorsolateral Amygdala (BLA/LaDL), Stratum (s.o.) oriens, (s.p.) pyramidale, (s.r.) radiatum, (s.l.m.) lacunosum-moleculare; granule cell layer (gcl).
